# Better BMI: Novel Digital Anthropometry Equations for Adiposity Assessment in the Chinese Population

**DOI:** 10.1007/s43657-026-00314-4

**Published:** 2026-04-01

**Authors:** Jialu Zhao, Xia Hu, Shujing Fu, Jialin Wang, Yan Zheng, Chen Chen, Jingchun Luo

**Affiliations:** 1 Human Phenome Institute, Zhangjiang Fudan International Innovation Center, Fudan University, Shanghai, 201203 China; 2 School of Life Sciences, Fudan University, Shanghai, 200438 China

**Keywords:** Digital anthropometry, Adiposity, BMI, Linear regression

## Abstract

The global obesity epidemic necessitates improved adiposity assessment alternatives beyond conventional anthropometric indices such as body mass index (BMI), particularly given their limitations in characterizing fat distribution. This study aimed to (1) develop adiposity assessment equations based on digital anthropometry and (2) systematically evaluate our equations against conventional anthropometric indices. We analyzed 1294 participants from the *International Human Phenome Project*, leveraging 151 digital anthropometric phenotypes and eight DXA-derived adiposity relative indices capturing adiposity from multiple distinct aspects. After training and comparing seven machine learning algorithms, the final equations were subjected to variance inflation factor (VIF) analysis to optimize predictor selection. A systematic evaluation was conducted, comparing the newly developed equations with both BMI and conventional alternative indices via Pearson correlation analysis. The forward stepwise linear regression (FSLR) model achieved superior or comparable accuracy ($R^2 = 0.767 - 0.910$) to the other six machine learning algorithms. Eight ethnicity-specific equations were derived via FSLR and VIF selection, incorporating 10.25 predictors on average. Systematic evaluation demonstrated that our equations outperformed BMI and conventional alternative indices by 2.3–63.0% across key adiposity metrics. Moreover, the results highlight the complementary value of combining BMI with alternative measures for comprehensive adiposity assessment. We developed adiposity assessment equations in the Chinese population using digital anthropometry, which significantly improved upon BMI-based evaluations. Furthermore, our findings provide concrete evidence for combining BMI with supplementary indices, as recently recommended by *The Lancet Diabetes & Endocrinology Commission*.

**Supplementary Information:**

The online version contains supplementary material available at 10.1007/s43657-026-00314-4.

## Introduction

The global prevalence of overweight and obesity has steadily increased in recent decades (NCD Risk Factor Collaboration 2016; NCD Risk Factor Collaboration 2017). Obesity is now widely recognized as a complex chronic disease and a major risk factor for numerous non-communicable diseases, including type 2 diabetes mellitus (T2DM), cardiovascular diseases (CVDs), and various types of cancer (Rubino et al. 2025; Cho et al. 2019; Lee et al. 2025; Lawrence and Kopelman 2004; Haslam and James 2005; Takahashi et al. 2006; The GBD 2015 Obesity Collaborators 2017). Notably, China currently has the highest number of overweight and obese individuals worldwide, with approximately half of all adults and one in five children affected (Wang et al. 2019, 2021).

Current anthropometric indices serve as accessible but limited proxies for adiposity assessment, including body mass index (BMI), waist circumference (WC), hip circumference (HC), neck circumference (NC), waist-to-hip ratio (WHR), waist-to-height ratio (WHtR), and a body shape index (ABSI), among others (Sweatt et al. 2024; Eknoyan 2007; Ashtary-Larky et al. 2018; Brambilla et al. 2013; Swainson et al. 2017; Krakauer and Krakauer 2012). BMI, defined as body weight divided by height squared, remains the most utilized indicator of adiposity in clinical and epidemiological settings due to its ease of calculation and broad recognition among researchers, clinicians, and the general public (Sweatt et al. 2024). However, BMI has several limitations when used as a surrogate measure of excess adiposity. For instance, BMI does not account for fat distribution and ignores the difference between fat and lean mass (Bray 2023; Ross et al. 2020; Janssen et al. 2004; Okorodudu et al. 2010). To overcome these limitations, alternative indices like WC have been developed to more accurately evaluate specific adiposity patterns, particularly central/visceral fat accumulation. *The Lancet Diabetes & Endocrinology Commission* has recommended direct adiposity measurements, such as dual-energy X-ray absorptiometry (DXA), or the incorporation of supplementary anthropometric criteria, including WC, WHR, and WHtR, alongside BMI (Rubino et al. 2025). Nevertheless, the approach for integrating BMI with other alternative indices in adiposity assessment remains unclear.

Direct methods for assessing adiposity include DXA, computed tomography (CT), and magnetic resonance imaging (MRI), among others (Earthman 2015; Teigen et al. 2017). These methods are constrained by high costs, technical complexity, and potential risks associated with radiation exposure (Goldman 2007; Teigen et al. 2017). CT and DXA scanners estimate body composition by measuring variations in X-ray attenuation; however, DXA offers significantly lower radiation exposure (Sweatt et al. 2024; Goldman 2007). In contrast to CT and DXA, MRI does not involve ionizing radiation. Nevertheless, its application in both clinical and research contexts is constrained by high operational costs and the need for specialized technical expertise (Earthman 2015). In summary, DXA is applicable to a broader range of clinical populations and is frequently used as a reference standard for validating other body composition assessment tools and predictive models (Goldman 2007; Teigen et al. 2017). Multiple studies have confirmed that DXA-derived metrics such as whole body fat mass (FM) (Choi et al. 2017; Skapino et al. 2025; Mohammad et al. 2023), fat mass index (FMI) (Skapino et al. 2025; Ofenheimer et al. 2020), whole body lean mass (LM) (Choi et al. 2017; Yuan et al. 2023), whole body fat percentage (BFP) (Wang et al. 2024a), android fat mass (Sari et al. 2019; Okosun et al. 2015; Min and Min 2015; Ma et al. 2023; Mohammad et al. 2023), gynoid fat mass (Okosun et al. 2015; Min and Min 2015; Ma et al. 2023; Mohammad et al. 2023), visceral adipose tissue mass (VAT) (Goto et al. 2024; Ofenheimer et al. 2020; Mohammad et al. 2023; Patel and Abate 2013), and the android-to-gynoid fat mass ratio (AGFMR) (Mohammad et al. 2023; Patel and Abate 2013; Aucouturier et al. 2009; Samsell et al. 2014) are significantly associated with various health conditions and disease risks. Nonetheless, like CT and MRI, DXA entails considerable cost, requires trained personnel, and due to its reliance on radiation, cannot be employed repeatedly in short time intervals.

As promising alternatives, emerging digital anthropometry technologies have garnered attention for indirectly evaluating body composition (Mocini et al. 2023; Porterfield et al. 2024) through mathematical models, which typically use DXA as the reference standard (Teigen et al. 2017). Estimates of body circumference derived from 3D optical scanners have demonstrated strong correlations with conventional manual methods, such as tape measurements (Pepper et al. 2010; Jaeschke et al. 2015). The accuracy of estimating fat-free mass (FFM) and FM has been reported to be comparable to that of DXA measurements (Lee et al. 2015, 2014; Bennett et al. 2022; Ng et al. 2016; Harty et al. 2020; Ng et al. 2019; Wong et al. 2019). Accurate prediction of regional body composition requires models developed from diverse and representative populations. However, existing studies using 3D imaging or anthropometric methods often suffer from limited sample sizes and narrow demographic coverage. For example, many studies include fewer than 200 participants (e.g., *n* = 121 Lee et al. 2015; *n* = 39 Ng et al. 2016), which may reduce statistical power and generalizability. Prior research frequently focuses on single-ethnic groups (e.g., European adults) or specific age ranges (e.g., children/adolescents), with limited inclusion of older adults, racial/ethnic minorities, or individuals with metabolic disorders (Lee et al. 2014; Harty et al. 2020; Ng et al. 2019; Wong et al. 2019). Therefore, larger sample size and more diverse ethnic groups are needed. Conversely, the proprietary prediction models (Thomas et al. 2025) exhibit significant variability across devices, and the lack of transparency in the underlying raw data and modeling approaches constrain the interpretability of the predictive mechanisms for non-technical users.

The primary objective of this study was to develop transparent mathematical equations with adiposity biomarkers to assess adiposity-related body composition indices using digital anthropometric phenotypes as input features, based on an extensive dataset of the Chinese population. A secondary objective was to systematically evaluate the proposed equations and conventional anthropometric indices in assessing adiposity relative indices. Furthermore, this study seeks to establish evidence-based recommendations on the standalone or combined use of alternative indices with BMI, thereby optimizing practical strategies for health status estimation.

## Materials and Methods

### Study Participants

This study undertook an analysis of multi-phenotype data from a cohort of 1434 participants recruited from China for the *International Human Phenome Project* (Phase I and II) in Shanghai, covering the period from May 2020 to January 2024. The Institutional Review Board (IRB) of the School of Life Sciences, Fudan University (Approval Nos. BE2048 and FE232741), reviewed and approved the research protocols. All participants provided written informed consent.

**Fig. 1:**
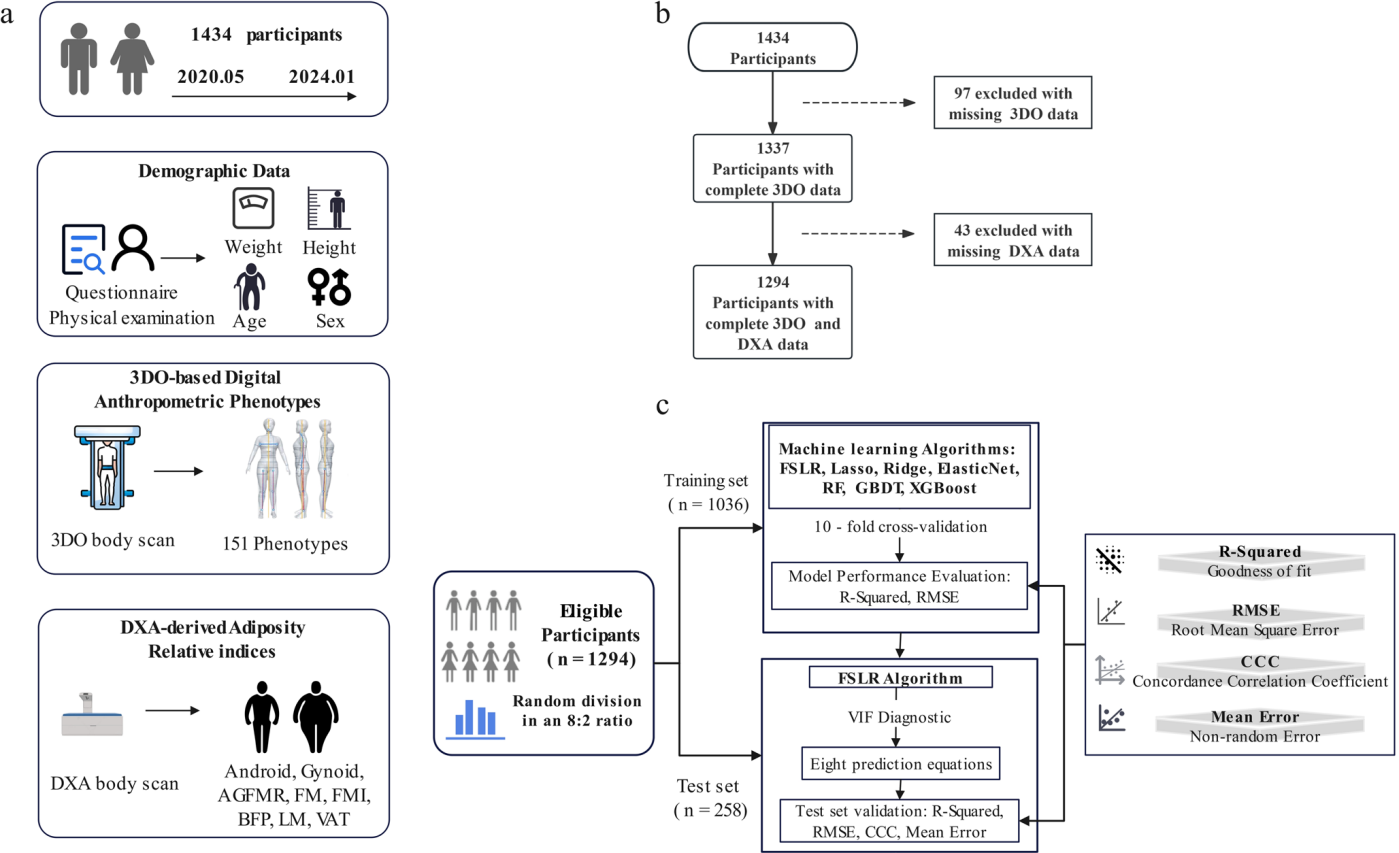
Overview of study design and participants. **a** Multi-dimensional data collection framework in the cohort study. **b** Flow chart of participant data preprocessing. **c** Development of equations based on digital anthropometric phenotypes

The inclusion criteria were as follows: (1) individuals aged 20–60 years with no intention to relocate from Shanghai within the next three years, (2) absence of major diseases, and (3) non-pregnant status. The cohort collected extensive phenotypic data, including demographic information, digital anthropometric phenotypes, and DXA-derived adiposity relative indices (Fig. 1a). 

The exclusion criteria were as follows: (1) participants with a missing rate greater than 5% for digital anthropometric phenotypes, or (2) participants with a missing rate greater than 5% for DXA-derived adiposity relative indices.

### Data Collection and Measurements

The study’s analysis encompassed demographic variables, including age, sex, height, and weight, as well as measurements obtained from three-dimensional optical (3DO) body scans and data derived from DXA scans. All measurements, encompassing both digital anthropometric phenotypes and body composition metrics, were conducted in accordance with standardized procedures and adhered to the manufacturer’s instructions for each respective device.

#### Demographic Data

All participants participated in face-to-face interviews utilizing questionnaires and physical examinations to gather sociodemographic data, including age, sex, height, and weight.

#### 3DO Body Scan Measurements

3DO body scans were conducted utilizing a laser triangulation technology-based system (Anthroscan BodyScan, Human Solutions GmbH, Germany; software version 3.6.2). This system consists of laser sensors, high-resolution cameras, a standardized scanning chamber, and specialized 3D analysis software. Participants were scanned while wearing tight-fitting clothing and swimming cap, maintaining a standardized posture (standing upright with feet placed on marked footprints, arms relaxed slightly away from the body, hands open, and head positioned in the Frankfurt plane). The Anthroscan automated measurement framework generates 151 digital anthropometric phenotypes through a machine-executable protocol converted from the ISO 7250 standards (Human Solutions GmbH, EP2867795B1, Claim 5).

This study assessed alternative body mass index (BMI, kg/m^2^ ) indices derived from anthropometric measurements, including waist circumference (WC, cm), hip circumference (HC, cm), and neck circumference (NC, cm). Additionally, the waist-to-hip ratio (WHR), the waist-to-height ratio (WHtR), and a body shape index (ABSI) were calculated, with the respective formulas provided below:

1 $$\begin{aligned} \text {WHR} = \frac{{\text {WC}}}{{\text {HC}}} \end{aligned}$$

2 $$\begin{aligned} \text {WHtR} = \frac{{\text {WC}}}{{\text {Height}}} \end{aligned}$$

3 $$\begin{aligned} \text {ABSI} = \frac{{\text {WC}}}{{\text {BMI}}^{2/3} \times \text {Height}^{1/2}} \end{aligned}$$

BMI was calculated as weight (*kg*) divided by height (*m*) squared (kg/m^2^). The BMI was evaluated according to the criteria established by the National Health Commission of China, as outlined in the national clinical practice guideline on obesity management (NHCotPRoc 2025). Participants were classified according to their BMI into the following categories: (a) underweight ( BMI < 18.5 kg/m^2^); (b) normal weight (18.5 *łe* BMI < 23.9 kg/m^2^); (c) overweight (24.0 *łe* BMI < 27.9 kg/m^2^); and (d) obese (*BMI **\ge* 28.0 kg/m^2^).

#### Dual-energy X-ray Absorptiometry (DXA) Scan Measurements

The DXA measurement was conducted using the Lunar iDXA (GE HealthCare, Inc., USA; software version Encore 13.60.033). DXA employs two X-ray sources with slightly different energy levels simultaneously, facilitating the differentiation and comparison of various tissue types, including bone, muscle, and fat. The device utilized in this study was approved in accordance with the local radiation protection agency, and a daily quality assessment was performed by certified radiology technicians.

All scans were administered by certified technicians with participants positioned supine, attired in light clothing and without footwear. The DXA scan offers a comprehensive evaluation of body composition, providing high-resolution quantification of total and regional body mass, adipose tissue distribution, lean soft tissue mass, bone mineral density, and other relevant indices. Out of 616 available datasets, eight adiposity-related indices were selected for analysis. These eight indices were selected to comprehensively characterize adiposity from multiple perspectives:

- Whole body fat mass (FM, kg): the total amount of adipose tissue in the human body.
- Whole body lean mass (LM, kg): the total body weight excluding fat, encompassing muscles, organs, bones, and other non-adipose tissues.
- Whole body fat percentage (BFP, %): the proportion of total body weight that is composed of fat mass.
- Android fat mass (Android, kg): the adipose tissue localized in the trunk region, particularly the abdominal area.
- Gynoid fat mass (Gynoid, kg): the fat mass in the hip and thigh regions.
- Visceral adipose tissue mass (VAT, kg): the total volume of fat depots surrounding internal organs within the abdominal cavity.
- Fat mass index (FMI, kg/m^2^): a body composition parameter derived by dividing whole-body fat mass by the square of height .
- Android-to-Gynoid fat mass ratio (AGFMR): the ratio of android fat mass to gynoid fat mass, reflecting the pattern of fat distribution across distinct anatomical regions.

### Data Preprocessing and Model Development

#### Data Preprocessing

Participants with more than *5%* missing data for either digital anthropometric phenotypes or DXA-derived adiposity relative indices were excluded from the study. Consequently, a total of 1294 participants were retained, following the exclusion of 97 individuals due to missing 3DO data and 43 individuals due to missing DXA measurements, respectively (Fig. 1b).

#### Model Comparison and Hyperparameter Optimization

The study population was randomly divided into training ( *n* = 1036 ) and test ( *n* = 258 ) sets in an 8:2 ratio. We trained and compared seven machine learning algorithms using the training dataset: forward stepwise linear regression (FSLR), lasso regression (Lasso), ridge regression (Ridge), elastic net regression (ElasticNet), random forest (RF), gradient boosted decision trees (GBDT), and eXtreme Gradient Boosting (XGBoost). The preprocessed dataset, comprising all 151 digital anthropometric phenotypes and demographic variables (sex, age, height, weight), served as input features for these machine learning models. These models were trained to predict eight adiposity relative indices: FM, FMI, LM, BFP, Android, Gynoid, VAT, and AGFMR.

To ensure the robustness of model performance and to rigorously prevent overfitting, this study utilized a nested cross-validation framework with hyperparameter tuning via random search for each model, based on a unified hyperparameter search space. A comprehensive hyperparameter search space was systematically defined for each machine learning algorithm.

For Lasso regression, the regularization strength *α* was explored across 100 logarithmically spaced values from $10^{-5}$ to $10^{2}$. Ridge regression employed a similar logarithmic grid spanning $10^{-3}$ to $10^{3}$ with equivalent resolution. Elastic Net optimization involved a two-dimensional search across 50 logarithmically spaced values for *α* (from $10^{-4}$ to $10^{2}$) combined with 10 linearly spaced L1-ratio values from 0.1 to 1.0, encompassing the continuum between L1 and L2 regularization.

Ensemble methods—including RF , GBDT and XGBoost—shared common architectural parameters: ensemble size (*n_estimators*: 50, 100, 200, 500), tree complexity (*max_depth*: 5, 10, 15, 20 for RF ; 3, 5, 7, 9 for GBDT/XGBoost), and regularization constraints. Gradient boosting implementations additionally incorporated learning rate variations (0.01, 0.05, 0.1, 0.2) and stochastic training parameters (*subsample*: 0.8 to 1.0 in 0.1 increments; *colsample_bytree*: 0.8 to 1.0 in 0.1 increments) to control overfitting and enhance generalization performance. All algorithms incorporated tree growth control parameters including minimum number of samples for node splitting (*min_samples_split*: 2, 5, 10) and minimum number of samples in leaf nodes (*min_samples_leaf*: 1, 2, 4).

Model training and evaluation were conducted via nested cross-validation. In the outer loop, a 10-fold cross-validation was used for performance evaluation, partitioning the dataset into 10 disjoint subsets, iteratively using one as the test set and the remaining nine as the training set to assess the final generalization performance. Furthermore, within the training set of each outer fold, a random search strategy was employed for hyperparameter optimization (inner loop). The random search sampled 10 parameter configurations from the predefined space and evaluated their performance using an inner 5-fold cross-validation (with negative mean-squared error as the metric). The best-performing configuration was then selected for the final model training on that outer fold. For the standard linear regression model, we employed sequential forward feature selection based on the Bayesian Information Criterion to build a parsimonious and interpretable model.

#### Model Performance Metrics

Model performance was first evaluated on the training set using 10-fold cross-validation with two metrics: coefficient of determination ($\text {R}^2$) and root mean square error (RMSE). The final model was assessed on the test set using four key metrics: $\text {R}^{2}$, RMSE, Mean Error (ME), and Concordance Correlation Coefficient (CCC). These metrics were selected to comprehensively assess the predictive accuracy, systematic bias, and overall model fit.

Specifically, RMSE measures the standard deviation of the residuals (prediction errors), providing an aggregate assessment of the model’s predictive accuracy. It was calculated as:

4 $$\begin{aligned} RMSE = \sqrt{\frac{1}{n}\sum _{i=1}^{n}(y_i - \hat{y}_i)^2} \end{aligned}$$

where $y_i$ represents the observed values, $\hat{y}_i$ denotes the predicted values, and *n* is the number of observations. A lower RMSE indicates better model performance.

ME was used to evaluate the systematic bias of the model, computed as the arithmetic mean of the prediction errors:

5 $$\begin{aligned} ME = \frac{1}{n}\sum _{i=1}^{n}(y_i - \hat{y}_i) \end{aligned}$$

A positive ME suggests an overall underestimation, whereas a negative ME indicates overestimation.

R^2^ quantifies the proportion of variance in the dependent variable that is predictable from the independent variables. The formula is:

6 $$\begin{aligned} R^2 = 1 - \frac{\sum _{i=1}^{n}(y_i - \hat{y}_i)^2}{\sum _{i=1}^{n}(y_i - \bar{y})^2} \end{aligned}$$

where $\bar{y}$ is the mean of the observed data. An R^2^ value close to 1 indicates a strong predictive relationship, while a value near 0 suggests poor model fit.

CCC measures the agreement between two variables, assessing not only linear correlation but also whether observed and predicted values fall along the 45^o^ line of perfect concordance. Its formula is:

7 $$\begin{aligned} CCC = \frac{2 \rho \sigma _x \sigma _y}{\sigma _x^2 + \sigma _y^2 + (\mu _x - \mu _y)^2} \end{aligned}$$

where *ρ* is the Pearson correlation coefficient (measuring linear relationship), $\mu _x$ and $\mu _y$ are the means of the two variables, and $\sigma _x^2$ and $\sigma _y^2$ are their variances.

The equation development process is illustrated in Fig. 1c.

#### Feature Importance Assessment

To systematically evaluate and compare feature importance, SHAP (SHapley Additive exPlanations) values were employed for analysis. SHAP, a method rooted in cooperative game theory, consistently breaks down model predictions into additive contributions from each feature. By examining the mean absolute SHAP value for each feature, a unified and reliable ranking of feature importance was established, reflecting the magnitude of each feature’s impact on the model’s output. Additionally, by exploring the relationship between feature values and their corresponding SHAP values, the direction of feature effects on predictions (whether positively or negatively correlated) can be interpreted. We integrated XGBoost modeling with SHAP visualization on FSLR-selected features to elucidate complex nonlinear patterns.

All computational analyses were performed using Python 3.11.4. The analytical workflow utilized the following software packages: numpy (1.24.3), pandas (1.5.3), scipy (1.10.1), scikit-learn (1.3.0), statsmodels (0.14.0), matplotlib (3.7.1), seaborn (0.12.2), and shap (0.48.0), all in Python.

### Statistical Analysis

Baseline characteristics were compared between the training and test sets using the chi-squared test for categorical variables and the *t*-test for continuous variables, contingent upon their distribution. Statistical significance was set at *p < 0.05* (two-tailed test). Differences in model performance between FSLR and the rest of machine learning algorithms were assessed using the Wilcoxon Signed-Rank test. We used a Bonferroni-adjusted significance threshold of *p < *8.3 *×* 10^-3 ^(*p* < 0.05/[6 model comparison] = 8.3 *×* 10^-3^) to determine statistical significance. FSLR algorithm selected predictors via the Bayesian Information Criterion (BIC), which was employed as the stopping rule to optimize the trade-off between model parsimony and explanatory power. To address multicollinearity, variables with variance inflation factors (VIF) exceeding 10 were excluded (O’brien 2007). The absence of severe autocorrelation was confirmed by Durbin–Watson test from the non-significant results (*p> 0.05*).

Normality was assessed using a comprehensive approach that integrated statistical testing with graphical analysis. The analysis focused on regression residuals, defined as the differences between observed and predicted values, whose normal distribution is a fundamental assumption for valid statistical inference in finite sample linear regression. The Kolmogorov-Smirnov goodness-of-fit test was employed to quantitatively evaluate normality, with a significance level of *α = 0.05* establishing that *p > 0.05* indicated no significant deviation from normality. Graphical assessment included Quantile-Quantile (Q-Q) plots, where alignment along the reference line suggested normality, complemented by calculation of the Q-Q correlation coefficient (values *> 0.98* indicating good normality). Distribution histograms with overlaid normal density curves provided visual representation of distribution shapes, while skewness (measuring distribution asymmetry, with values near zero indicating symmetry) and kurtosis (quantifying tail heaviness relative to the normal distribution, with values near 3 representing mesokurtic distributions) offered additional descriptive metrics. This multi-faceted evaluation protocol was systematically applied to regression residuals, dependent variables, and key independent variables across all models using a consistent sample size of *n* = 1036 .

Bland–Altman agreement analysis was utilized to assess the validity of the developed equations, visualizing the mean difference and *95%* limits of agreement between DXA measurements and predicted values. To compare the accuracy of different algorithms in assessing adiposity relative indices, Pearson correlation coefficients (*r*) were calculated between each anthropometric method (both conventional and digital) and eight DXA-derived adiposity relative indices. The interpretation of Pearson correlation coefficients was stratified using multi-tiered criteria adapted from contemporary biomarker research guidelines (Steyerberg 2019): coefficients with absolute values *\ge*0.85 were classified as *Very strong* correlations, values between 0.7–0.85 denoted *Strong* correlations, correlations of 0.5–0.7 indicated *Moderate* correlations, while values below 0.5 represented *Weak* correlations. This classification system was fundamentally aligned with biological plausibility boundaries rather than statistical significance levels (Koterov et al. 2019).

All computational analyses were performed using Python 3.11.4 and R 4.3.2. The analytical workflow utilized the following software packages: numpy (1.24.3), pandas (1.5.3), scipy (1.10.1), scikit-learn (1.3.0), statsmodels (0.14.0), matplotlib (3.7.1) in Python; alongside tidyverse (2.0.0), dplyr (1.1.4), tidyr (1.3.1), broom (1.0.9), car (3.1.3), lmtest (0.9.40), sandwich (3.1.1), moments (0.14.1), nortest (1.0.4), tseries (0.10.58), ggplot2 (3.5.2), patchwork (1.3.2), and caret (7.0.1) in R.

## Results

### Baseline Characteristics

The analytical cohort comprised 1294 participants (female-to-male ratio of 1.1:1) who had complete 3DO and DXA data. This cohort was randomly divided into training and test sets in an 8:2 ratio. Baseline characteristics are detailed in Table 1. The mean age was 34.3 years (range: 20–63), with *99.7%* of the participants aged 20–60. Four justified exceptions (61–63 years) resulted from COVID-related delays (baseline data collected at ages 58–60 years). Regarding BMI, *4.71%* of the population was classified as underweight, *59.81%* as normal weight, *26.74%* as overweight, and *8.73%* as obese. No significant differences in demographic or anthropometric measures (e.g., age, height, weight, BMI) were observed between the training and validation sets (*p> 0.05* for all).

**Table 1:** Baseline characteristics of participants in the training and test sets

Variable	Overall (*n* = 1294)	Training set (*n* = 1036)	Test set (*n* = 258)	$\boldsymbol{p}$
Gender				0.18
Female	678 (52.40%)	533 (51.45%)	145 (56.20%)	
Male	616 (47.60%)	503 (48.55%)	113 (43.80%)	
Age (y)	34.30 ± 11.26	34.12 ± 11.16	35.02 ± 11.64	0.26
Height (m)	1.66 ± 0.09	1.66 ± 0.08	1.66 ± 0.09	0.24
Weight (kg)	64.27 ± 12.01	64.55 ± 12.12	63.12 ± 11.5	0.08
BMI (kg/m^2^)	23.19 ± 3.40	23.25 ± 3.44	22.93 ± 3.2	0.35
BMI Categories				0.35
Underweight	61 (4.71%)	47 (4.54%)	14 (5.43%)	
Normal	774 (59.81%)	612 (59.07%)	162 (62.79%)	
Overweight	346 (26.74%)	280 (27.03%)	66 (25.58%)	
Obesity	113 (8.73%)	97 (9.36%)	16 (6.20%)	
FM (kg)	19.06 ± 6.18	19.19 ± 6.18	18.53 ± 6.13	0.12
FMI (kg/m^2^)	6.94 ± 2.27	6.98 ± 2.26	6.79 ± 2.29	0.26
LM (kg)	42.65 ± 8.59	42.8 ± 8.65	42.04 ± 8.31	0.19
BFP (%)	29.57 ± 6.88	29.65 ± 6.85	29.26 ± 7.00	0.42
Android (kg)	1.53 ± 0.79	1.55 ± 0.79	1.45 ± 0.74	0.06
Gynoid (kg)	3.11 ± 0.98	3.12 ± 0.98	3.05 ± 1.00	0.29
VAT (kg)	0.63 ± 0.52	0.64 ± 0.53	0.59 ± 0.50	0.15
AGFMR	0.49 ± 0.18	0.49 ± 0.19	0.47 ± 0.18	0.12
WC (cm)	78.48 ± 9.86	78.7 ± 9.94	77.6 ± 9.48	0.10
HC (cm)	95.49 ± 5.74	95.62 ± 5.70	94.96 ± 5.89	0.10
NC (cm)	35.06 ± 3.67	35.16 ± 3.70	34.66 ± 3.54	0.06
WHR	0.82 ± 0.07	0.82 ± 0.07	0.82 ± 0.07	0.29
WHtR	0.47 ± 0.06	0.47 ± 0.06	0.47 ± 0.06	0.25
ABSI	0.07 ± 0.004	0.07 ± 0.004	0.07 ± 0.004	0.48

Categorical variables were presented as frequencies (percentages). Continuous variables were presented as mean ± standard deviation. The *p-*value was calculated using *t*-tests (for continuous variables) or chi-square tests (for categorical variables)

Sex-specific differences in adiposity measures are presented in Table S1. The mean age of male participants was 34.58 years (range: 20–60), while that of female participants was 33.99 years (range: 20–63). No statistically significant age difference was observed between the sexes (*p = 0.35*). However, significant sex differences were noted in height, body weight, and BMI categories ($p < 1\times 10^{-4}$). Although the results for FM were not significant (*p = 0.30*), significant sex-based disparities were identified in other DXA-derived adiposity relative indices ($p < 1\times 10^{-4}$).

### Comparison of Seven Machine Learning Algorithms on the Training Set

**Fig. 2:**
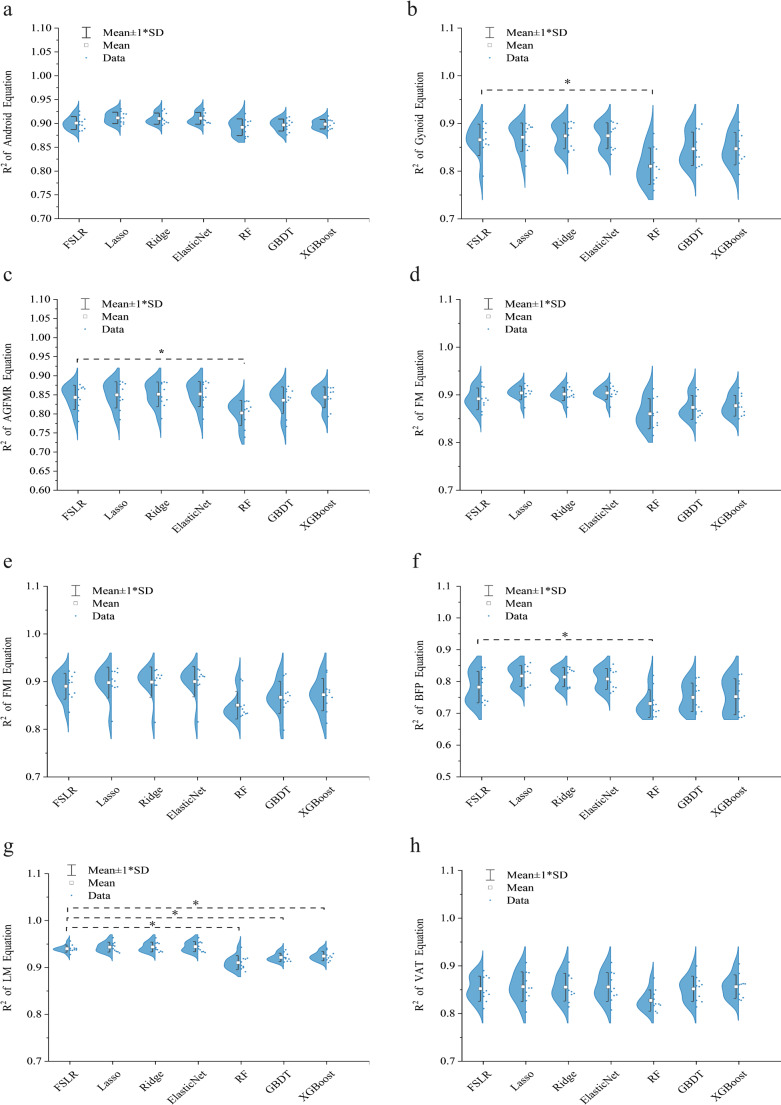
Comparison of FSLR performance metrics with other machine learning algorithms. Violin and box plots for $\textbf{R}^2$ of all machine learning algorithms for all adiposity relative indices. The *p-*value was calculated using the Wilcoxon rank–sum test. * indicates statistical significance (p < 8.3 × 10^-3^ )

**Fig. 3:**
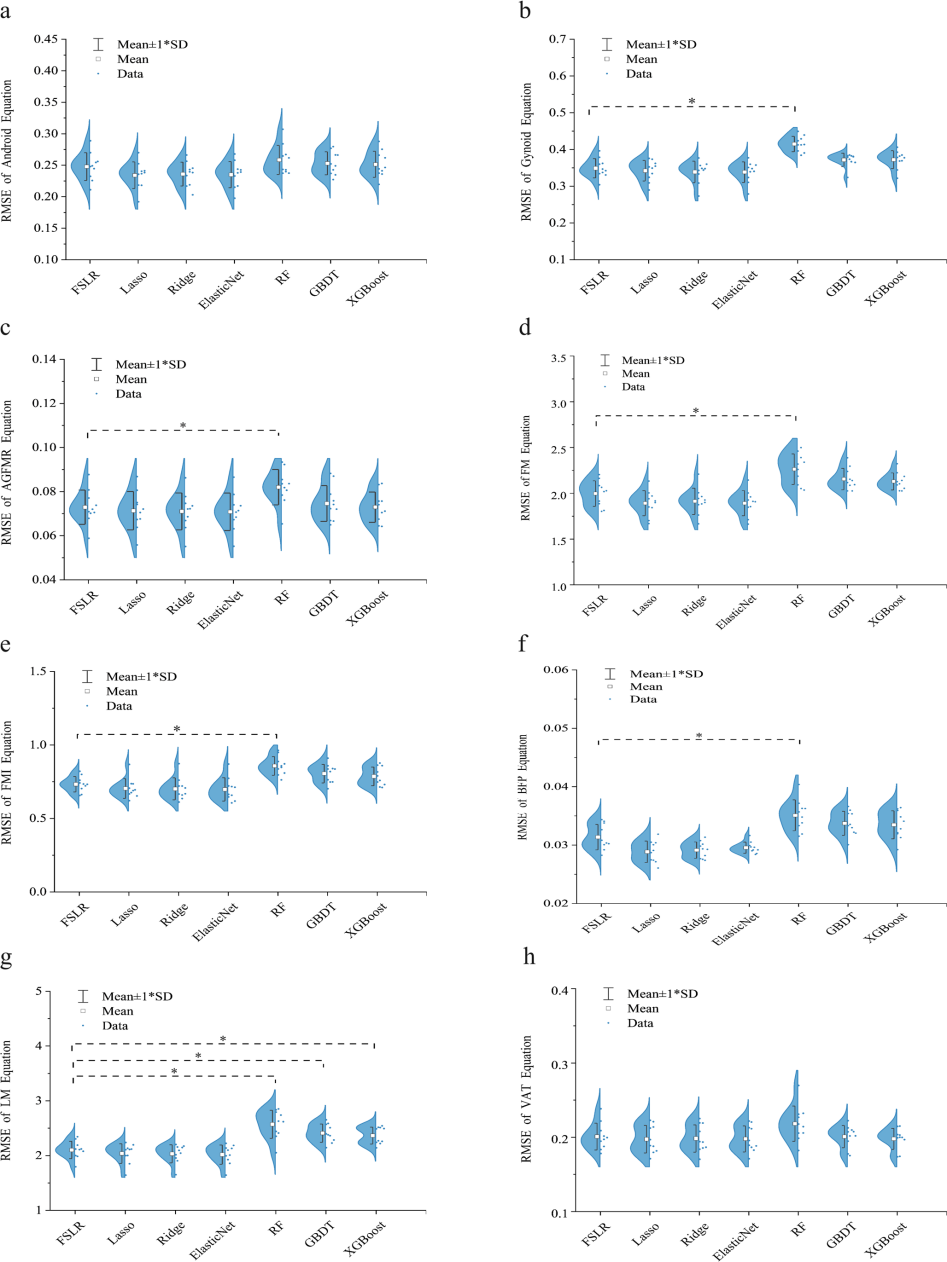
Comparison of FSLR performance metrics with other machine learning algorithms. Violin and box plots for **RMSE** of all machine learning algorithms for all adiposity relative indices. The *p*-value was calculated using the Wilcoxon rank–sum test. * indicates statistical significance ( *p* < 8.3 × 10^-3^)

Table 2 summarizes the performance metrics, specifically RMSE and R^2^, for seven predictive algorithms—FSLR, Lasso, Ridge, ElasticNet, RF, GBDT, and XGBoost—employing digital anthropometric phenotypes and demographic indicators within the training set (*80%*, *n* = 1036 ). The results indicate that the four algorithms—FSLR, Lasso, Ridge, and ElasticNet—exhibited lower predictive errors (lower RMSE) and higher variance explanation (higher R^2^) compared to the others. Pairwise comparisons based on R^2^ and RMSE indicated that none of the machine learning algorithms employed in this study demonstrated statistically significant superiority over FSLR (Figs. 2, 3). Notably, FSLR exhibited enhanced predictive accuracy relative to RF, GBDT, and XGBoost in forecasting LM (Figs. 2g, 3g). Similarly, FSLR exceeded RF in predicting Gynoid, AGFMR and BFP (Fig. 2b,c,f, Fig. 3b,c,f). For specific outcomes such as Android and VAT, no significant differences were observed that would suggest the rest of the algorithms were superior to FSLR.

Due to its robust performance in multivariate adjustments ($R^2 \ge 0.78$) and its mathematical transparency facilitated by stepwise feature selection criteria, FSLR was chosen for the primary analysis (Harrell 2015; Heinze et al. 2018). The FSLR framework offers mechanistic clarity by explicitly linking input variables to outcomes, thereby allowing clinicians to audit decision pathways (Rudin 2019). This is consistent with the requirements of translational research, where model interpretability and operational traceability are essential for ethical validation (Steyerberg 2019).

**Table 2:** Performance metrics of the machine learning algorithms on the training set

Metrics	Machine Learning Models						
	FSLR	Lasso	Ridge	ElasticNet	RF	GBDT	XGBoost
**Android**							
$R^2$	0.90	0.91	0.91	0.91	0.89	0.90	0.90
RMSE	0.25	0.23	0.24	0.24	0.26	0.25	0.25
**Gynoid**							
$R^2$	0.87	0.87	0.87	0.87	0.81	0.85	0.85
RMSE	0.35	0.34	0.34	0.34	0.42	0.37	0.37
**AGFMR**							
$R^2$	0.84	0.85	0.85	0.85	0.80	0.84	0.84
RMSE	0.07	0.07	0.07	0.07	0.08	0.08	0.07
**FM**							
$R^2$	0.89	0.90	0.90	0.90	0.86	0.87	0.88
RMSE	2.00	1.89	1.89	1.91	2.26	2.16	2.13
**FMI**							
$R^2$	0.89	0.90	0.90	0.90	0.85	0.87	0.87
RMSE	0.73	0.70	0.70	0.70	0.86	0.80	0.79
**BFP**							
$R^2$	0.78	0.82	0.81	0.81	0.73	0.75	0.75
RMSE	0.03	0.03	0.03	0.03	0.04	0.03	0.03
**LM**							
$R^2$	0.94	0.94	0.94	0.94	0.91	0.92	0.92
RMSE	2.11	2.04	2.02	2.03	2.57	2.41	2.37
**VAT**							
$R^2$	0.85	0.86	0.86	0.86	0.83	0.85	0.86
RMSE	0.20	0.20	0.20	0.20	0.22	0.20	0.20

### Prediction Equations Based on Digital Anthropometric Phenotypes via FSLR Algorithms

The finalized prediction equations, incorporating unstandardized regression coefficients (*β*), are presented in Table 3. The selection of predictors was conducted using VIF diagnostics, with a threshold of VIF *\ge 10*, within the FSLR framework. This process reduced the initial set of 155 predictor candidates to 8 –16 variables per model, with a mean of 10.25. All retained predictors demonstrated statistical significance (*p < 0.05*) in the final equations. Sex consistently emerged as a predictor across all regression equations, with the exception of the AGFMR model, while age was included in models specifically predicting Gynoid, FM, FMI, and VAT. Ankle girth was universally included, except in the Gynoid and VAT mass prediction equation. Notably, waist girth, forearm girth, calf girth, and knee girth were frequently selected ($\ge 50\%$ occurrence) during stepwise model iteration. Body weight was excluded from variable selection due to multicollinearity (VIF *> 10*). Variance decomposition analysis indicated that the retained anthropometric variables across all equations collectively accounted for an average of 96.4 % of weight variance in the validation cohort (*n* = 258) (Table S2). The non-significant Durbin-Watson tests ($\text {DW} = {1.85~to~2.04}$,*p> 0.05*) suggest no substantial residual autocorrelation in our models. This finding supports the critical assumption of independent errors, confirming that our regression estimates are unbiased and statistically efficient irrespective of data ordering (Table S3).

Regression residuals conformed to normality assumptions (Kolmogorov-Smirnov test: *p* > 0.05 ; Q-Q correlation *> 0.98*), which validated the outcomes of FSLR models. Residual normality results are detailed in Table S4, with diagnostic plots in Fig. S1. Dependent and key independent variables predominantly exhibited non-normal distributions (Table S5), visualized in Fig. S2.

SHAP values were computed utilizing the XGBoost model, incorporating corresponding features to predict eight adiposity-related indices employed in our predictive equations. The summary plots, as depicted in Fig. S3, reveal that the primary influential features vary across different models. Anthropometric indicators such as waist girth, sex, body height, and age demonstrate differing levels of significance in each model. Moreover, changes in features like sex and waist girth exert distinct directional effects on the model outputs, with the degree of variation in impact amplitude differing markedly among various features.

Furthermore, stratified validation by sex and age groups was conducted for all prediction equations that demonstrated robust performance across all subgroups ($R^2 \ge 0.48$), with the exception of the equation related to VAT. Notably, the initial VAT equation exhibited poor predictive accuracy in young women aged 20–30 years ($R^2 = -0.14$). To address the stratification effects of age and sex, interaction terms between waist circumference and demographic variables (age and sex) were incorporated into the final VAT equation. This modification resulted in enhanced predictive performance ($R^2 = 0.43$) in the 20–30-year-old female subgroup while maintaining strong accuracy across all other strata. Detailed results of the stratified analysis are provided in Fig. S4.

**Table 3:** Derived prediction equations from digital anthropometric phenotypes (Part 1 of 2)

**Outcome**	Predictors	$\boldsymbol{\beta }$	$\boldsymbol{p}$
Android (kg)	Intercept	−4.949	$<10^{-4}$
	Sex	−0.207	$<10^{-4}$
	Ankle girth left (cm)	−0.044	$<10^{-4}$
	Arm length left (cm)	−0.018	$<10^{-4}$
	Forearm girth right (cm)	−0.032	$<10^{-4}$
	Hip/thigh girth (cm)	−0.011	$<10^{-4}$
	Knee girth left (cm)	0.058	$<10^{-4}$
	Waist girth (cm)	0.086	$<10^{-4}$
	Waist to buttock left (cm)	0.077	$<10^{-4}$
Gynoid (kg)	Intercept	−6.475	$<10^{-4}$
	Age (year)	−0.004	0.0066
	Sex	0.640	$<10^{-4}$
	Across back width (armpit level, cm)	−0.021	$<10^{-4}$
	Calf girth right (cm)	−0.056	$<10^{-4}$
	Crotch length rear (cm)	0.048	$<10^{-4}$
	Forearm girth right (cm)	−0.067	$<10^{-4}$
	High hip girth (cm)	0.064	$<10^{-4}$
	Knee girth left (cm)	0.090	$<10^{-4}$
	Thigh girth right (horizontal, cm)	0.105	$<10^{-4}$
	Upper arm length left (cm)	−0.026	0.0016
	Width armpits (cm)	−0.024	$<10^{-4}$
AGFMR	Intercept	0.043	0.4100
	Ankle girth left (cm)	−0.008	$<10^{-4}$
	Bust/chest girth (cm)	0.005	$<10^{-4}$
	Forearm length left (cm)	−0.004	0.0006
	Hip girth (cm)	−0.009	$<10^{-4}$
	Mid neck girth (cm)	0.011	$<10^{-4}$
	Neck to across back width (armpit level, cm)	0.004	0.0046
	Thigh girth left (horizontal, cm)	−0.012	$<10^{-4}$
	Waist girth (cm)	0.016	$<10^{-4}$
	Waist to high hip back (cm)	0.015	$<10^{-4}$
FM (kg)	Intercept	−45.529	$<10^{-4}$
	Age (year)	−0.054	$<10^{-4}$
	Sex	−6.559	$<10^{-4}$
	Ankle girth left (cm)	−0.463	$<10^{-4}$
	Calf girth left (cm)	−0.125	0.0270
	Crotch length (cm)	0.269	$<10^{-4}$
	Forearm girth right (cm)	−0.212	0.0008
	Head height (cm)	−0.195	0.0046
	Knee girth left (cm)	0.612	$<10^{-4}$
	Min leg girth left (cm)	0.482	$<10^{-4}$
	Waist girth (cm)	0.534	$<10^{-4}$
FMI (kg/m^2^)	Intercept	−4.344	$<10^{-4}$
	Age (year)	−0.010	0.0004
	Sex	−0.258	0.0049
	Ankle girth right (cm)	−0.072	0.0011
	Arm length left (cm)	−0.068	$<10^{-4}$
	Body height (cm)	−0.064	$<10^{-4}$
	Buttock girth (cm)	0.227	$<10^{-4}$
	Knee girth left (cm)	0.181	$<10^{-4}$
	Upper arm girth right (cm)	0.055	$<10^{-4}$

**Table 4:** Derived prediction equations from digital anthropometric phenotypes (Part 2 of 2)

Outcome	Predictors	$\boldsymbol{\beta }$	$\boldsymbol{p}$
BFP (%)	Intercept	19.625	$<10^{-4}$
	Sex	−6.999	$<10^{-4}$
	Across back width (armpit level, cm)	−0.221	$<10^{-4}$
	Ankle girth left (cm)	−0.646	$<10^{-4}$
	Arm length left (cm)	−0.180	$<10^{-4}$
	Calf girth left (cm)	−0.587	$<10^{-4}$
	Crotch length rear (cm)	0.237	0.0003
	Distance neck to buttock (cm)	0.317	$<10^{-4}$
	Forearm girth right (cm)	−0.624	$<10^{-4}$
	Head height (cm)	−0.374	0.0005
	Hip/thigh girth (cm)	−0.206	$<10^{-4}$
	Knee girth left (cm)	0.708	$<10^{-4}$
	Min leg girth left (cm)	0.749	$<10^{-4}$
	Neck left to waist back (cm)	−0.450	$<10^{-4}$
	Thigh girth right (horizontal, cm)	0.378	$<10^{-4}$
	Waist girth (cm)	0.600	$<10^{-4}$
	Width armpits (cm)	−0.128	$<10^{-4}$
LM (kg)	Intercept	−65.993	$<10^{-4}$
	Sex	4.509	$<10^{-4}$
	Ankle girth left (cm)	0.375	$<10^{-4}$
	Body height (cm)	0.321	$<10^{-4}$
	Calf girth left (cm)	0.799	$<10^{-4}$
	Forearm girth right (cm)	1.072	$<10^{-4}$
	High hip girth (cm)	−0.048	0.0016
	Knee girth left (cm)	−0.155	0.0067
	Min leg girth left (cm)	−0.347	0.0054
	Width armpits (cm)	0.137	$<10^{-4}$
VAT (kg)	Intercept	0.342	0.1330
	Age (year)	−0.046	$<10^{-4}$
	Arm length right (cm)	−0.012	$<10^{-4}$
	Sex	−1.585	$<10^{-4}$
	Thigh girth left (horizontal, cm)	−0.019	$<10^{-4}$
	Waist girth (cm)	0.018	$<10^{-4}$
	Waist to high hip back (cm)	0.044	$<10^{-4}$
	Age (year) *×* Waist girth (cm)	0.0006	$<10^{-4}$
	Sex *×* Waist girth (cm)	0.021	$<10^{-4}$

Sex: 0 is for female; 1 is for male

### Validation for the Prediction Equations for DXA-derived Adiposity Relative Indices

The prediction equations developed for DXA-derived adiposity relative indices were validated using the test set (20 %, *n* = 258) to evaluate cross-validation. As presented in Table 5, all 95% confidence intervals for mean prediction errors (observed − predicted values) encompassed zero, indicating no statistically significant differences between DXA-measured and equation-predicted values (all *p> 0.05*). The validation results demonstrated strong correlations with DXA reference values for most indices (Table 5): Android ($R^2 = 0.878$), Gynoid ($R^2 = 0.861$), AGFMR ($R^2 = 0.806$), FM ($R^2 = 0.873$), FMI ($R^2 = 0.878$), LM ($R^2 = 0.910$) and VAT ($R^2 = 0.813$). Moderately strong correlations were observed for BFP ($R^2 = 0.767$).

**Fig. 4:**
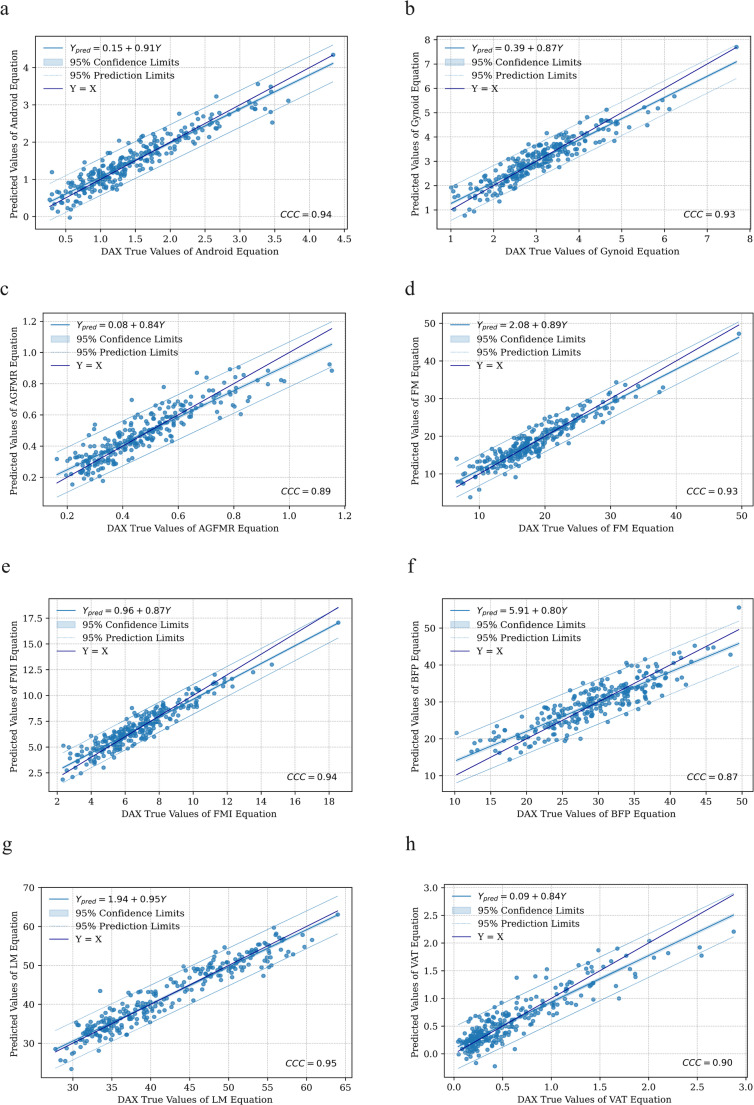
Regression scatterplots for DXA values vs. predicted values derived from prediction equations on the test set. CCC, Concordance Correlation Coefficient

Linear regression plots, depicted in Fig. 4, illustrate the predicted adiposity indices against DXA measurements. These plots demonstrate that the estimates from the equations achieved exceptional concordance with DXA measurements, exhibiting a high level of agreement for all body adiposity indices (CCC: 0.94 for Android, 0.93 for Gynoid, 0.89 for AGFMR, 0.93 for FM, 0.94 for FMI, 0.87 for BFP, 0.95 for LM, and 0.90 for VAT).

**Fig. 5:**
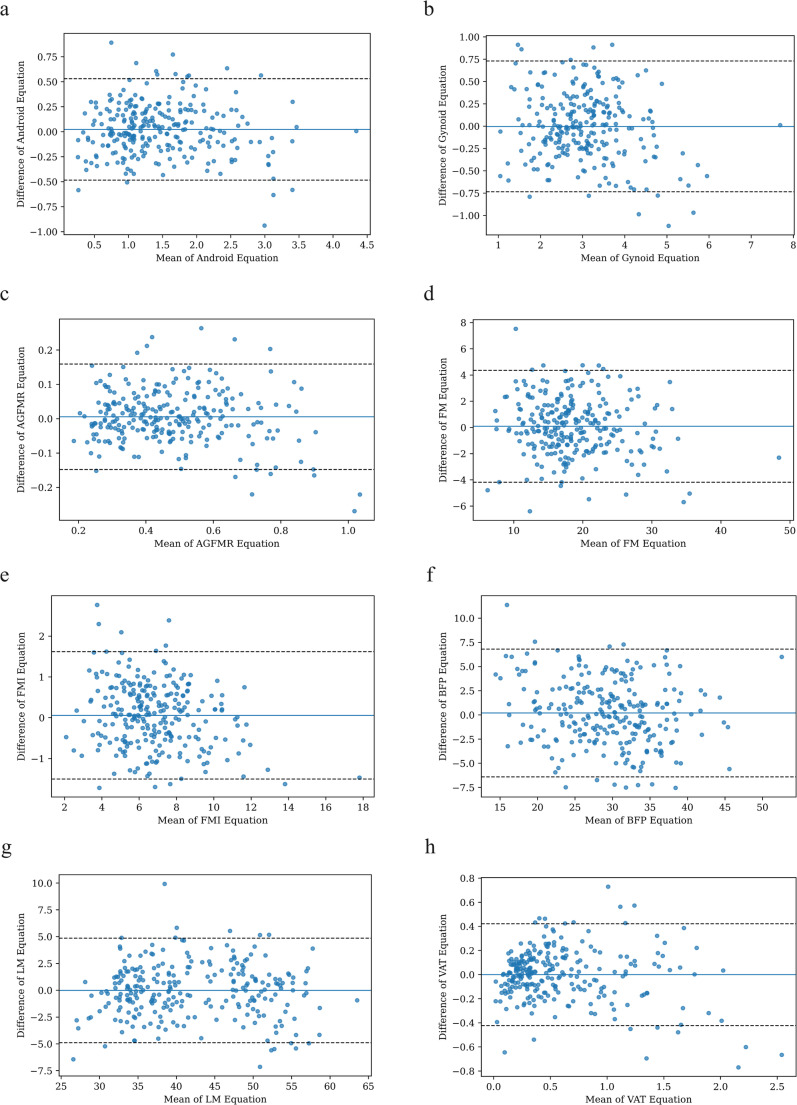
Bland-Altman plots for DXA values vs. predicted values derived from prediction equations on the test set

Figure 5 displays the mean difference (solid line) along with the *95%* agreement limits (dotted lines) obtained from Bland-Altman analysis, which assesses potential systematic bias between DXA-derived adiposity relative indices and equation-predicted values. The data points were uniformly distributed within the *95%* agreement range, showing no discernible trends, thereby supporting the reliability of the predictive equations. The limits of agreement for Android and Gynoid adiposity distribution were *0.023 ± 0.507* kg for Android, *-0.003 ± 0.732* kg for Gynoid, and *0.006 ± 0.153* for AGFMR. The limits of agreement for total body adiposity distribution were *0.084 ± 4.271* kg for FM, *0.056 ± 1.562* kg/m^2^ for FMI, and $0.193\% \pm 6.602\%$ for BFP. The limits of agreement for LM were *-0.023 ± 4.878* kg, while the limits of agreement for VAT were *0.001 ± 0.422* kg. The random dispersion of data points within agreement limits, along with the absence of systematic error patterns and only sporadic outliers attributable to biological variation rather than measurement error, collectively validate the robustness of these equations for adiposity assessment.

**Table 5:** Performance metrics of prediction equations on the training and test sets

Outcome	Training Set			Test Set		
	$R^2$	RMSE	ME (95% CI)	$R^2$	RMSE	ME (95% CI)
Android	0.885	0.269	0.000 (−0.016, 0.016)	0.878	0.260	0.023 (−0.009, 0.055)
Gynoid	0.866	0.357	0.000 (−0.022, 0.022)	0.861	0.374	−0.003 (−0.048, 0.043)
AGFMR	0.844	0.073	0.000 (−0.004, 0.004)	0.806	0.078	0.006 (−0.004, 0.015)
FM	0.877	2.170	0.000 (−0.132, 0.132)	0.873	2.181	0.084 (−0.184, 0.352)
FMI	0.868	0.823	0.000 (−0.050, 0.050)	0.878	0.799	0.056 (−0.041, 0.154)
BFP	0.787	3.160	0.000 (−0.193, 0.193)	0.767	3.374	0.193 (−0.220, 0.607)
LM	0.921	2.429	0.000 (−0.148, 0.148)	0.910	2.489	−0.023 (−0.329, 0.283)
VAT	0.848	0.207	0.000 (−0.013, 0.013)	0.813	0.215	−0.001 (−0.027, 0.026)

ME (Mean error) = observed - predicted (95% confidence interval)

### Systematic Evaluation in Assessment of DXA-derived Adiposity Relative Indices

As illustrated in Table 6 and Table S6, our multivariate predictive equations exhibited significantly improved correlations (ranging from 2.3% to 63.0% enhancement; all *|r| \ge 0.88*, *p < 0.0001*) when compared to conventional anthropometric methods across all DXA-derived adiposity relative indices. The BMI demonstrated robust correlations with Android (*r = 0.85*), FM (*r = 0.82*), FMI (*r = 0.77*), and VAT (*r = 0.72*), surpassing clinical screening accuracy thresholds. Notable correlations were identified between BMI and Gynoid (*r = 0.62*), as well as the AGFMR (*r = 0.60*), aligning with moderate clinical relevance thresholds according to current standards. Importantly, BMI correlations with BFP (*r = 0.46*) and LM (*r = 0.48*) were classified as weak correlations (all *p < 0.0001*). WC exhibited very strong correlation magnitude with Android (*r = 0.90*) and VAT (*r = 0.85*), outperforming BMI. In comparison, HC showed a strong correlation with Gynoid (*r = 0.78*), while a very strong correlation was observed between WHtR and Android (*r = 0.85*). The WHR demonstrated very strong correlations with AGFMR (*r = 0.88*) and VAT (*r = 0.81*). Notably, NC exhibited superior predictive capacity as a strong and clinically significant correlation of LM (*r = 0.81*) compared to conventional anthropometric indices, suggesting its potential as a novel biomarker.

**Table 6:**
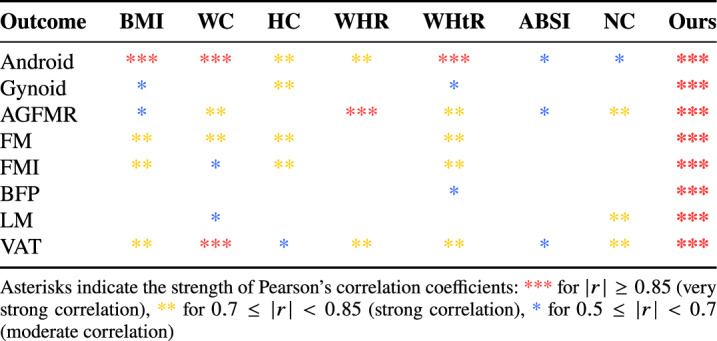
Systematic evaluation of our equations and conventional anthropometric indices in the assessment of adiposity relative indices

## Discussion

This study introduces a transparent mathematical framework for the development of interpretable prediction equations of DXA-derived adiposity relative indices utilizing digital anthropometric phenotypes. Our findings indicate that FSLR algorithms achieve predictive accuracy comparable to or exceeding that of complex machine learning algorithms, while retaining mathematical transparency—a crucial advantage for clinical application. The validated equations exhibited exceptional precision in predicting DXA-derived adiposity relative indices, as evidenced by high concordance correlation coefficients (CCC *≥ 0.87* across all indices) and minimal systematic bias. Furthermore, systematic quantitative comparisons using Pearson’s correlation coefficients revealed stronger correlations between our predictive equations and DXA-derived adiposity relative indices (*r = 0.88 - 0.94*), surpassing those of conventional anthropometric indices by 2.3%–63.0%. These findings provide empirical support for the earlier recommendations proposed by *The Lancet Diabetes & Endocrinology Commission* (Rubino et al. 2025), which emphasize the necessity of incorporating supplementary anthropometric measures in conjunction with BMI.

In line with *The Lancet Diabetes & Endocrinology Commission*’s call for improved obesity assessment, our systematic comparison provides empirical support for the necessity of integrating supplementary anthropometric measures with BMI to achieve a more comprehensive evaluation. While BMI maintains utility for population-level screening with moderate correlations across all adiposity relative indices (mean *r=0.67*), other anthropometric substitutions yield significant enhancements. WC demonstrates stronger correlations with Android than BMI (*Δ r=+0.05*), whereas HC exhibits a *26%* higher sensitivity to Gynoid compared to BMI (*Δ r=+0.16*). WHR significantly surpasses BMI in AGFMR assessment (*Δ r=+0.28*), and WC again shows an advantage in VAT quantification (*Δ r=+0.13*). Notably, NC demonstrates a *65%* improvement in LM correlation over BMI (*Δ r=+0.33*), establishing its independent evaluation value. Furthermore, our correlation analysis (Table 6.) establishes a systematic framework for selecting anthropometric indices and optimizing measurement strategies for adiposity assessment, demonstrating our equations’ superior accuracy compared to BMI. The combined use of BMI with specific anthropometric measures significantly enhances evaluation precision: WC or WHtR improves Android assessment, HC refines Gynoid quantification, WHR strengthens AGFMR evaluation, WC advances VAT estimation, and NC optimizes LM assessment. This integrated approach provides a comprehensive framework for evaluating adiposity relative indices.

Our research advances previous studies by introducing several significant enhancements. Firstly, our study, which encompasses a cohort of 1294 Chinese individuals (678 females and 616 males) aged 20–63, significantly broadens the scope of existing research that often focuses on smaller and less diverse groups, such as those in Lee et al. (2015, 2014) and Harty et al. (2020). This larger and more representative sample enhances the reliability and applicability of our findings to the broader Chinese population, addressing a critical gap in the literature regarding ethnic diversity. The balanced gender ratio and broad age range of our cohort enhance the generalizability of our findings, contrasting with studies like Wong et al. (2019), which focused on younger age groups. Additionally, by concentrating on a single ethnic group, we avoid the potential confounding factors observed in multi-ethnic studies such as Bennett et al. (2022) and Ng et al. (2016, 2019). Secondly, unlike black-box algorithms that rely on raw 3D point cloud computations (Ng et al. 2019; Qiao et al. 2024), our use of ISO-standardized measurement protocols (e.g., ankle girth left) provides clinically meaningful length and circumference parameters. This standardized approach not only ensures cross-platform compatibility with diverse 3D scanning technologies but also maintains applicability to conventional manual measurement techniques, thereby significantly enhancing methodological translatability and clinical adoption potential. Thirdly, our variable selection process, utilizing the FSLR framework and VIF diagnostics, reduced the initial 155 phenotypes to 8–16 core predictors without sacrificing accuracy. Notably, when employing stratified validation by sex and age groups, the initial VAT equation demonstrated limited predictive accuracy in young women aged 20–30 years. We hypothesized that this stratification effect arises because young women, in contrast to other subgroups, typically exhibit a fat distribution pattern that is more subcutaneous than visceral, as previously reported (Ethun 2016; Blaak 2001). This finding underscores the critical importance of considering demographic effect modifiers in the development of predictive body composition models to ensure their applicability across the diverse physiological realities of the population.

Our findings should be interpreted with consideration of three limitations. First, the derivation cohort’s strict focus on Chinese adults mainly aged 20–60 years intentionally excluded validation in pediatric, geriatric, and multi-ethnic populations. This selectivity limits immediate generalizability to groups with distinct adiposity patterns, such as the elderly exhibiting sarcopenic obesity (Prado et al. 2024) or ethnic cohorts with visceral fat redistribution, as extensively documented in South Asian populations (Lesser et al. 2012). Second, our systematic evaluation of conventional anthropometric indices intentionally excluded skinfold thickness assessment, as its measurement precision critically depends on operator-dependent landmark identification—a well-documented source of variability (coefficient of variation *\ge*15% in multi-center trials) as per NIH Body Composition Working Group standards (Protocol BC-0025-R4). Third, the participants in this study were exclusively recruited from a single metropolitan area, specifically Shanghai, China. Given that populations from different regions exhibit substantial variations in genetic background, dietary habits, and lifestyle factors, the generalizability of the prediction equations developed in this study to other regions of China may be limited. Therefore, caution is warranted when applying these equations to distinct populations.

In specific scenarios, 3D scanning technology offers a more suitable alternative to DXA for assessing fat distribution. Applications include body composition analysis in fitness and health sculpting (Balasubramanian and Sheykhmaleki 2025), rehabilitation therapy (Lu et al. 2025), and population-based health surveys (Wang et al. 2024b). Compared to DXA, 3D scanning is non-ionizing, more cost-effective, and allows for frequent repeated assessments—making it particularly advantageous for vulnerable groups (e.g., pregnant women and children) as well as long-term monitoring studies (Balasubramanian and Sheykhmaleki 2025; Börgeson et al. 2024; Tian et al. 2023; Vozgoment et al. 2023; Mettler 2023; Kamiya et al. 2015). Our method overcomes the constraints of traditional 3D body scanning (Thomas et al. 2025), making it possible the development of significantly simpler and more affordable 3D measurement devices. By reducing the required input to just 8–16 key anthropometric measurements, we bypass the computational complexity and opacity of conventional body fat prediction models, while maintaining comparable accuracy.

The equations developed using adiposity biomarkers facilitate cost-effective and radiation-free evaluation of body composition in accordance with global initiatives to overcome the limitations of BMI (Bray 2023). Future research should prioritize the independent external validation of these predictive equations in more diverse cohorts, taking into account distinct genetic, dietary, and lifestyle factors.

## Conclusion

This study establishes novel mathematical equations for adiposity assessment using digital anthropometric phenotypes as biomarkers, demonstrating that FSLR achieves comparable or superior predictive accuracy to complex machine learning approaches. Our key contributions include: (1) development of predictive equations using adiposity biomarkers for 1294 Chinese adults, addressing the critical gap in ethnicity-specific adiposity assessment; and (2) systematic evaluation revealing our equations’ superiority and providing evidence-based indices combinations to complement BMI for comprehensive evaluation. Future research should focus on multi-center validation across diverse populations and clinical outcome correlations to establish prognostic utility.

## Supplementary Information

Below is the link to the electronic supplementary material.

Supplementary file 1 (pdf 21044 KB)

## Data Availability

The data utilized in this study were obtained from the International Human Phenome Project. All data are accessible through the PhenoBank repository (https://www.phenobank.org.cn). Applications for data access should be submitted via https://www.phenobank.org.cn/apply.

## Abbreviations

3DO: Three dimensional optical
ABSI: A body shape index
AGFMR: Android-to-gynoid fat mass ratio
Android: Android fat mass
BFP: Whole body fat percentage
BMI: Body mass index
CCC: Concordance correlation coefficient
DXA: Dual-energyx-ray Absorptiometry
ElasticNet: Elastic net regression
FM: Whole body fat mass
FMI: Fat mass index
FSLR: Forward stepwise linear regression
GBDT: Gradient boosted decision tree
Gynoid: Gynoid fat mass
HC: Hip circumference
Lasso: Least absolute shrinkage and selection operator regression
LM: Whole body lean mass
ME: Mean error
NC: Neck circumference
RF: Random forest
Ridge: Ridge regression
RMSE: Root mean square error
SHAP: Shapley additive explanations
VAT: Visceral adipose tissue mass
VIF: Variance inflation factor
WC: Waist circumference
WHtR: Waist-to-height ratio
WHR: Waist-to-hip ratio
XGBoost: Extreme gradient boosting

## Declarations

### Code Availability

The custom code employed for data preprocessing, model training, feature selection, statistical analysis, and figure generation in this study has been deposited in the following public repository: https://github.com/zhaojialu520/Better_BMI_Code.
