# Supplementary material for: Better BMI: Novel Digital Anthropometry Equations for Adiposity Assessment in the Chinese Population
Source: Phenomics. 2026 Apr 1;6(2):117–36. doi: 10.1007/s43657-026-00314-4 (PMC13481917; doi:10.1007/s43657-026-00314-4)
Supplement: Supplementary file 1 [file 43657_2026_314_MOESM1_ESM.pdf]

Better BMI: Novel Digital Anthropometry Equations for Adiposity Assessment in Chinese Population

Jialu Zhao[1], Xia Hu[1], Shujing Fu[1], Jialin Wang[1], Yan Zheng[1,2], Chen Chen[1], **Jingchun Luo**[1*]

[1*] Human Phenome Institute, Zhangjiang Fudan International Innovation Center, Fudan University Shanghai, 201203, China.
[2]School of Life Sciences, Fudan University, Shanghai 200438, China.

*Corresponding author(s). E-mail(s): luojc@fudan.edu.cn; ORCID: 0000-0001-5138-6458;
Contributing authors: 23212030025@m.fudan.edu.cn; 24112030011@m.fudan.edu.cn; fushujing@fudan.edu.cn; wangjialin@fudan.edu.cn; yan_zheng@fudan.edu.cn; chenchen_fd@fudan.edu.cn;

1 Supplementary Information

Table S 1: General description of the participants' baseline characteristics.

Variable[1]	Females (N=678)	Males (N=616)	p value [2]
Age (years)	34.58 ± 11.44	33.99 ± 11.05	0.35
Height (m)	1.60 ± 0.05	1.72 ± 0.07	$< 10^{-4}$
Weight (kg)	57.4 ± 8.58	71.82 ± 10.63	$< 10^{-4}$
BMI (kg/m^2)	22.3 ± 3.15	24.17 ± 3.39	$< 10^{-4}$
BMI Categories			$< 10^{-4}$
Underweight	44 (6.49%)	17 (2.76%)	
Normal	470 (69.32%)	304 (49.35%)	
Overweight	128 (18.88%)	218 (35.39%)	
Obesity	36 (5.31%)	77 (12.50%)	
FM (kg)	19.23 ± 5.74	18.87 ± 6.62	0.30
FMI (kg/m^2)	7.48 ± 2.21	6.35 ± 2.19	$< 10^{-4}$
AGFMR	0.39 ± 0.13	0.59 ± 0.18	$< 10^{-4}$
Android (kg)	1.37 ± 0.67	1.71 ± 0.86	$< 10^{-4}$
Gynoid (kg)	3.41 ± 0.9	2.78 ± 0.96	$< 10^{-4}$
VAT (kg)	0.44 ± 0.36	0.83 ± 0.6	$< 10^{-4}$
LM (kg)	35.9 ± 3.87	50.08 ± 5.75	$< 10^{-4}$
BFP (%)	33.03 ± 5.49	25.76 ± 6.22	$< 10^{-4}$
WC (cm)	74.31 ± 8.75	83.08 ± 8.92	$< 10^{-4}$
HC (cm)	94.5 ± 5.7	96.58 ± 5.59	$< 10^{-4}$
NC (cm)	32.41 ± 2.18	37.98 ± 2.61	$< 10^{-4}$
WHR	0.79 ± 0.06	0.86 ± 0.06	$< 10^{-4}$
WHtR	0.46 ± 0.06	0.48 ± 0.05	$< 10^{-4}$
ABSI	0.74 ± 0.04	0.76 ± 0.04	$< 10^{-4}$

[1]Categorical variables are presented as frequency (percentage). Continuous variables are presented as mean ± standard deviation.

[2]***p*** values were calculated using t-tests (for continuous variables) or chi-square tests (for categorical variables).

Table S 2: Model-specific predictor counts and weight variance explained in digital anthropometric regression models.

Models	Retained Predictors (n)	Weight Variance Explained (%)
Android Model	8	96.90
Gynoid Model	11	96.43
AGFMR Model	9	96.40
FM Model	10	95.34
FMI Model	8	96.79
BFP Model	16	97.70
LM Model	9	95.73
VAT Model	6	95.93

Table S 3: Predictors selected by VIF and results from the DW test (Part 1 of 2).

Outcome	Predictors	VIF[1]	DW[2]	*p* value
Android (kg)	Sex	4.90	1.967	0.296
	Ankle Girth Left (cm)	2.99		
	Arm length left (cm)	2.39		
	Forearm girth right (cm)	4.81		
	Hip/thigh girth (cm)	2.97		
	Knee girth left (cm)	3.17		
	Waist girth (cm)	3.60		
	Waist to buttock left (cm)	1.46		
Gynoid (kg)	Age (year)	1.89	2.037	0.578
	Sex	4.53		
	Across back width (armpit level, cm)	2.76		
	Calf girth right (cm)	4.86		
	Crotch length rear (cm)	2.60		
	Forearm girth right (cm)	5.17		
	High hip girth (cm)	4.02		
	Knee girth left (cm)	3.48		
	Thigh girth right (horizontal, cm)	5.95		
	Upper arm length left (cm)	2.91		
	Width armpits (cm)	1.46		
AGFMR	Ankle girth left (cm)	2.54	1.849	0.070
	Bust/chest girth (cm)	6.12		
	Forearm length left (cm)	1.31		
	Hip girth (cm)	6.72		
	Mid neck girth (cm)	5.03		
	Neck to across back width (armpit level, cm)	1.65		
	Thigh girth left (horizontal, cm)	5.95		
	Waist girth (cm)	8.17		
	Waist to high hip back (cm)	1.28		
FM (kg)	Age (year)	1.84	1.921	0.099
	Sex	4.24		
	Ankle girth left (cm)	4.97		
	Calf girth left (cm)	5.74		
	Crotch length (cm)	5.09		
	Forearm girth right (cm)	5.10		
	Head height (cm)	1.76		
	Knee girth left (cm)	3.38		
	Min leg girth left (cm)	5.65		
	Waist girth (cm)	4.22		
FMI (kg/m^2)	Age (year)	1.43	1.992	0.444
	Sex	3.15		
	Ankle girth right (cm)	3.04		
	Arm length left (cm)	4.49		
	Body height (cm)	4.88		
	Buttock girth (cm)	3.04		
	Knee girth left (cm)	2.97		
	Upper arm girth right (cm)	2.30		

Continued on next page

Table S 3: Predictors selected by VIF and results from the DW test (Part 2 of 2)

**Outcome**	**Predictors**	**VIF[1]**	**DW[2]**	***p* value**
	Sex	3.92	1.922	0.101
	Across back width (armpit level, cm)	3.00		
	Ankle girth left (cm)	5.15		
	Arm length left (cm)	3.09		
	Calf girth left (cm)	6.76		
	Crotch length rear (cm)	3.12		
	Distance neck to buttock (cm)	6.02		
BFP (%)	Forearm girth right (cm)	5.43		
	Head height (cm)	2.00		
	Hip/thigh girth (cm)	6.52		
	Knee girth left (cm)	4.08		
	Min leg girth left (cm)	5.72		
	Neck left to waist back (cm)	5.54		
	Thigh girth right (horizontal, cm)	9.26		
	Waist girth (cm)	5.31		
	Width armpits (cm)	1.60		
	Sex	3.55	2.012	0.578
	Ankle girth left (cm)	4.90		
	Body height (cm)	2.43		
	Calf girth left (cm)	5.52		
LM (kg)	Forearm girth right (cm)	4.57		
	High hip grith (cm)	2.77		
	Knee girth left (cm)	3.38		
	Min leg girth left (cm)	5.52		
	Width armpits (cm)	1.36		
	Age (year)	1.70	1.899	0.051
	Arm length right (cm)	1.39		
	Thigh girth left (horizontal, cm)	2.98		
	Upper arm girth left (cm)	3.64		
VAT (kg)	Waist girth (cm)	3.78		
	Waist to high hip back (cm)	1.14		

[1]VIF, Variance Inflation Factor.

[2]DW, Durbin-Watson test.

Table S 4: Kolmogorov-Smirnov normality test results of the residuals for all models.

**Model**	***p* value**
Android Model	0.3026
Gynoid Model	0.3915
A_G Model	0.5518
FM Model	0.0564
FMI Model	0.9788
BFP Model	0.6898
LM Model	0.9222
VAT Model	0.1060

Note: K-S test examines the null hypothesis that data follow normal distribution. P-values < 0.05 indicate significant deviation from normality.

Table S 5: Kolmogorov-Smirnov normality test results of the dependent and key independent variables.

**Variable**	**p value**	**Variable**	***p* value**
FMI (kg/m^2)	0.0126	Buttock girth (cm)	0.0020
A_G	0.0009	Body height (cm)	0.0015
FM (kg)	0.0017	Ankle girth right (cm)	0.0164
LM (kg)	$< 10^{-4}$	Arm length left (cm)	0.0573
VAT (kg)	$< 10^{-4}$	Upper arm girth right (cm)	0.1218
Android (kg)	$< 10^{-4}$	High hip girth (cm)	0.0006
Gynoid (kg)	0.0016	Width armpits (cm)	**0.6694**
BFP (%)	**0.0942**	Hip/thigh girth (cm)	0.0275
Waist girth (cm)	0.0288	Waist to buttock left (cm)	**0.5637**
Thigh girth left (horizontal, cm)	0.0051	Thigh girth right (horizontal, cm)	0.0028
Waist to high hip back (cm)	**0.5596**	Calf girth right (cm)	0.0033
Arm length right (cm)	**0.0630**	Across back width (armpit level, cm)	0.0045
Age (year)	$< 10^{-4}$	Crotch length rear (cm)	0.0102
Sex	$< 10^{-4}$	Upper arm length left (cm)	0.0005
Knee girth left (cm)	0.0021	Hip girth (cm)	0.0035
Ankle girth left (cm)	0.0042	Mid neck girth (cm)	$< 10^{-4}$
Forearm girth right (cm)	0.0038	Bust/chest girth (cm)	0.0114
Crotch length (cm)	0.0009	Neck to across back width (armpit level, cm)	**0.0896**
Head height (cm)	**0.0862**	Forearm length left (cm)	**0.1928**
Calf girth left (cm)	0.0033	Neck left to waist back (cm)	**0.2716**
Min leg girth left (cm)	0.0033	Distance neck to buttock (cm)	**0.1119**

Note: Sex is coded as 0 for female and 1 for male. The Kolmogorov-Smirnov (K-S) test is employed to assess the null hypothesis that the data conform to a normal distribution. P-values less than 0.05 indicate a significant deviation from normality. Variables that are consistent with a normal distribution ($P \geq 0.05$) are highlighted in boldface.

Table S 6: Pearson's correlation coefficients between alternative anthropometric indices and DXA-derived adiposity relative indices.

**Outcome**	**BMI**	**WC**	**HC**	**WHR**	**WHtR**	**ABSI**	**NC**	**Ours**
Android	0.85	0.90	0.75	0.71	0.85	0.50	0.64	0.94
Gynoid	0.62	0.46	0.78	0.08	0.50	0.01	0.10	0.93
AGFMR	0.60	0.81	0.34	0.88	0.72	0.66	0.76	0.90
FM	0.82	0.75	0.83	0.44	0.74	0.27	0.42	0.93
FMI	0.77	0.64	0.72	0.38	0.77	0.25	0.25	0.94
BFP	0.46	0.36	0.49	0.16	0.54	0.22	-0.08	0.88
LM	0.48	0.57	0.48	0.45	0.23	0.07	0.81	0.95
VAT	0.72	0.85	0.50	0.81	0.79	0.60	0.72	0.90

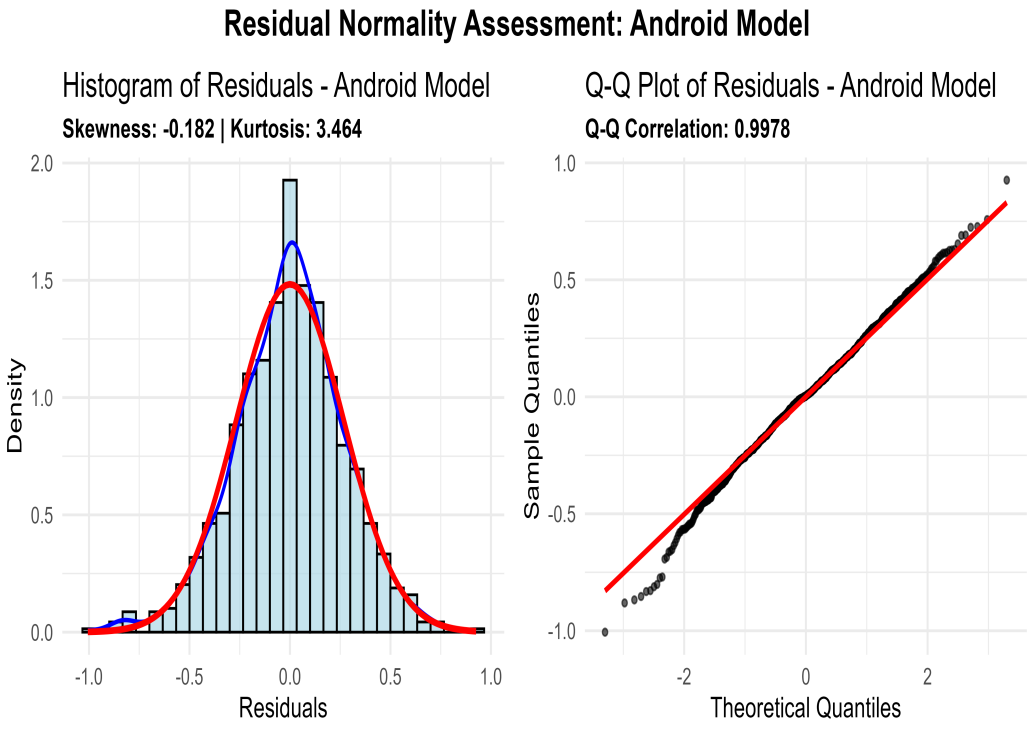

(a)

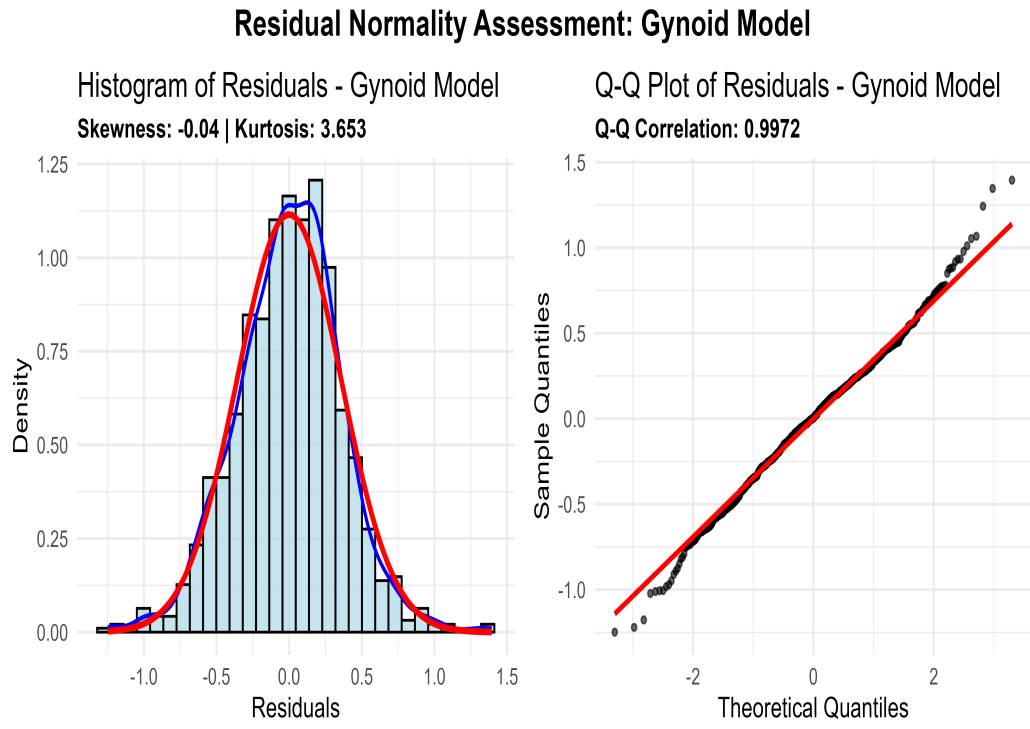

(b)

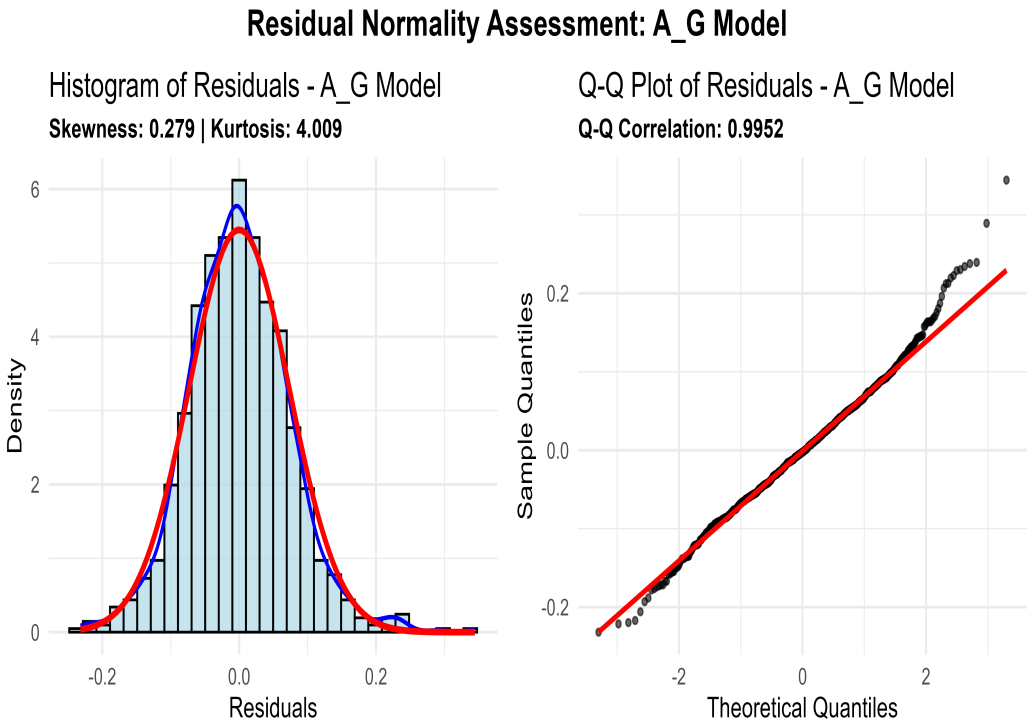

(c)

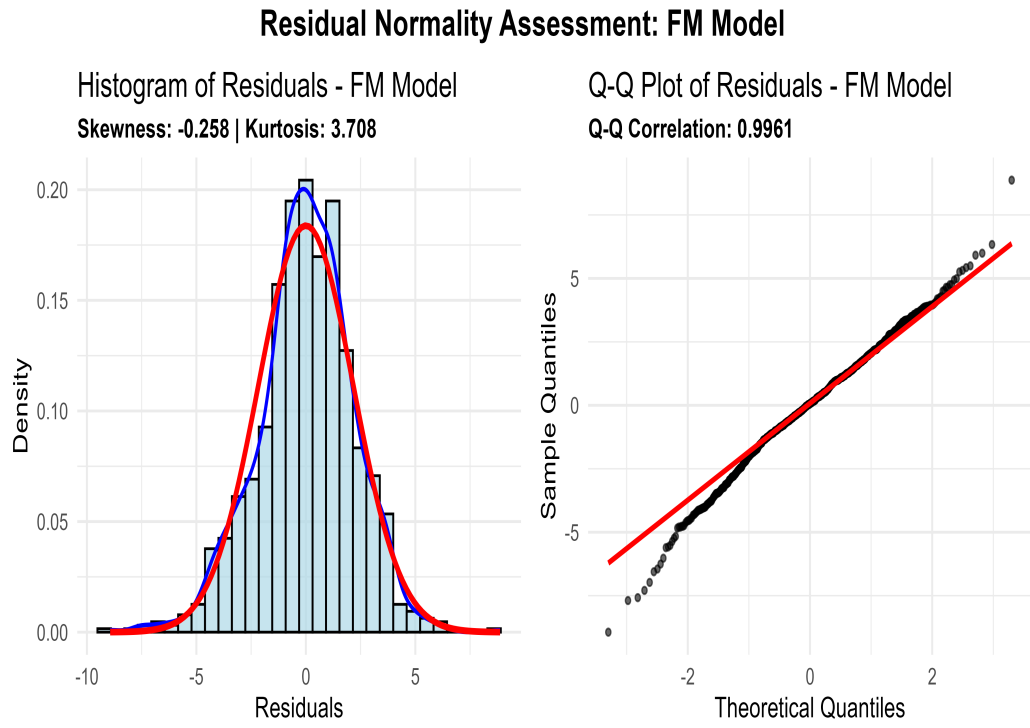

(d)

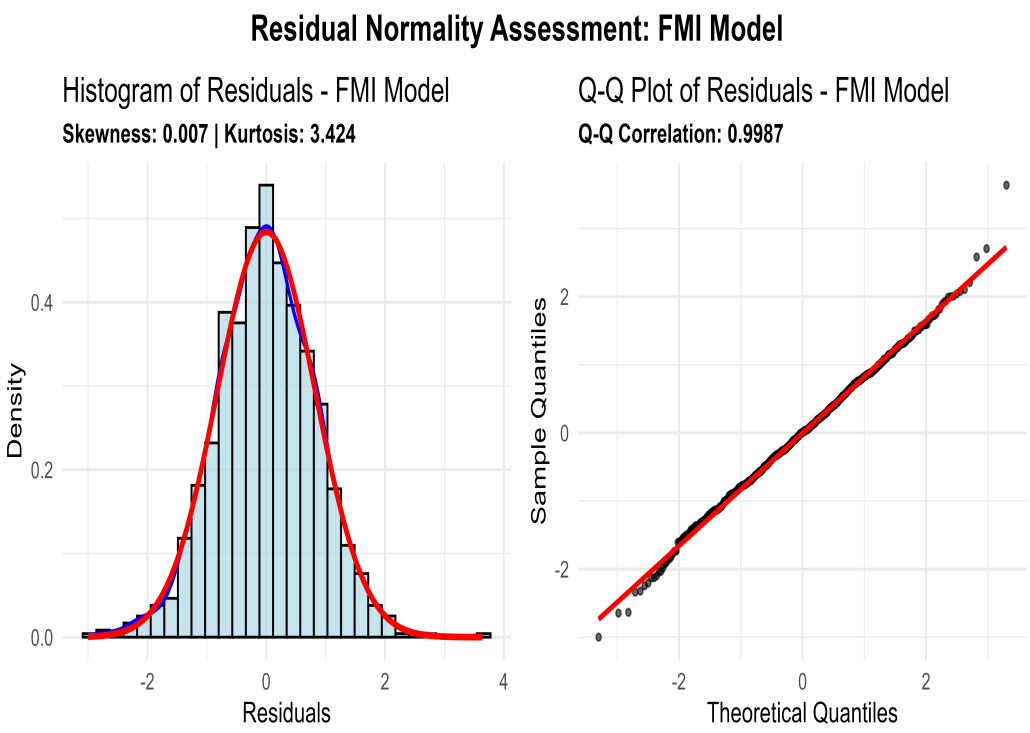

(e)

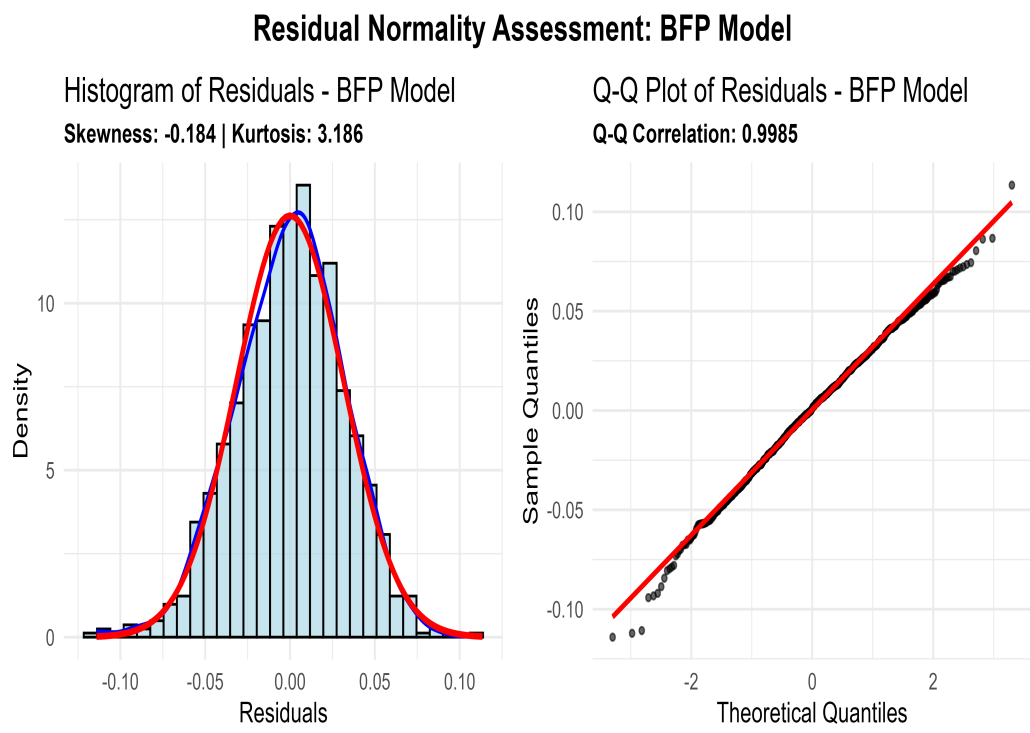

(f)

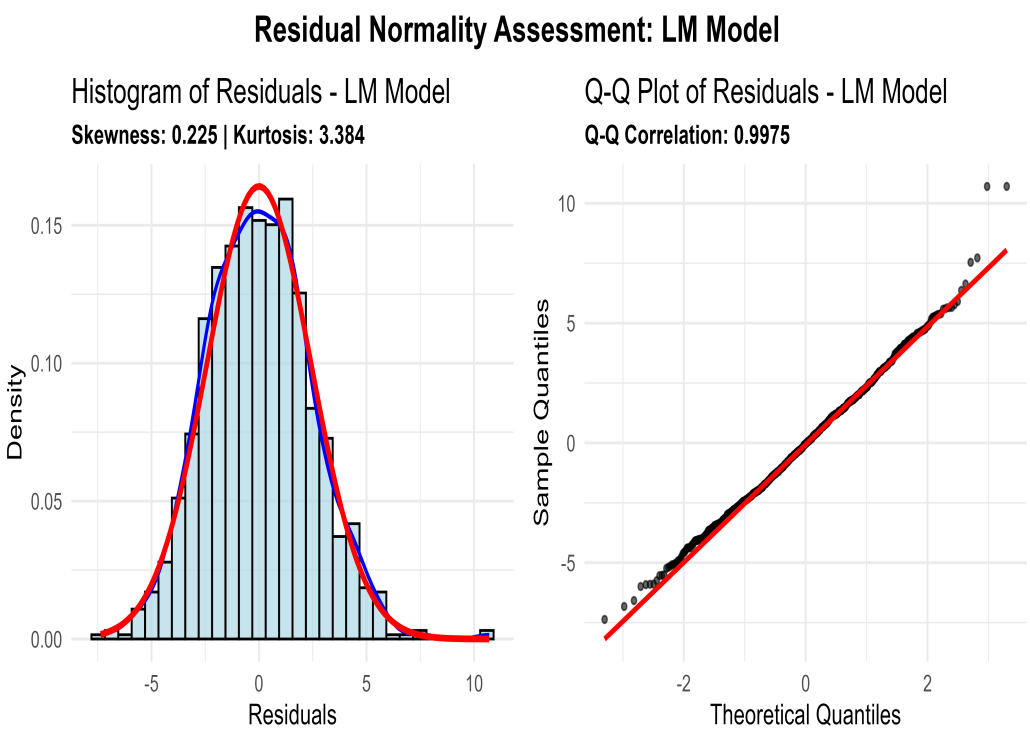

(g)

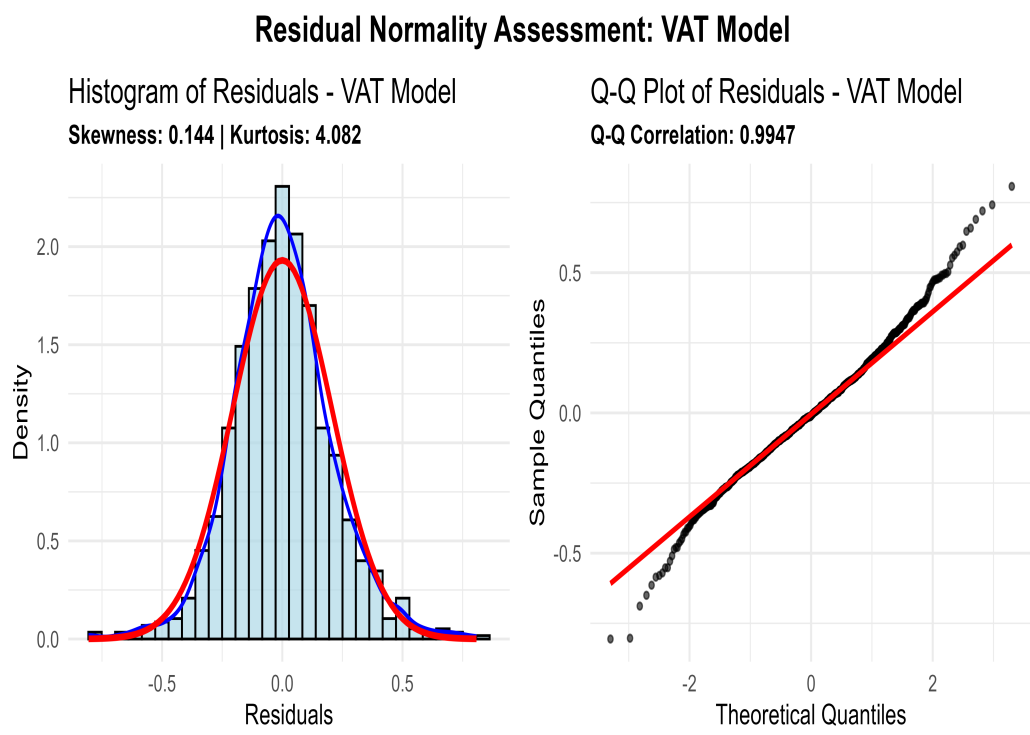

(h)

Fig. S 1: Residual Quantile-Quantile (Q-Q) Plots and Histograms for Different FSLR Models (N = 1,036).

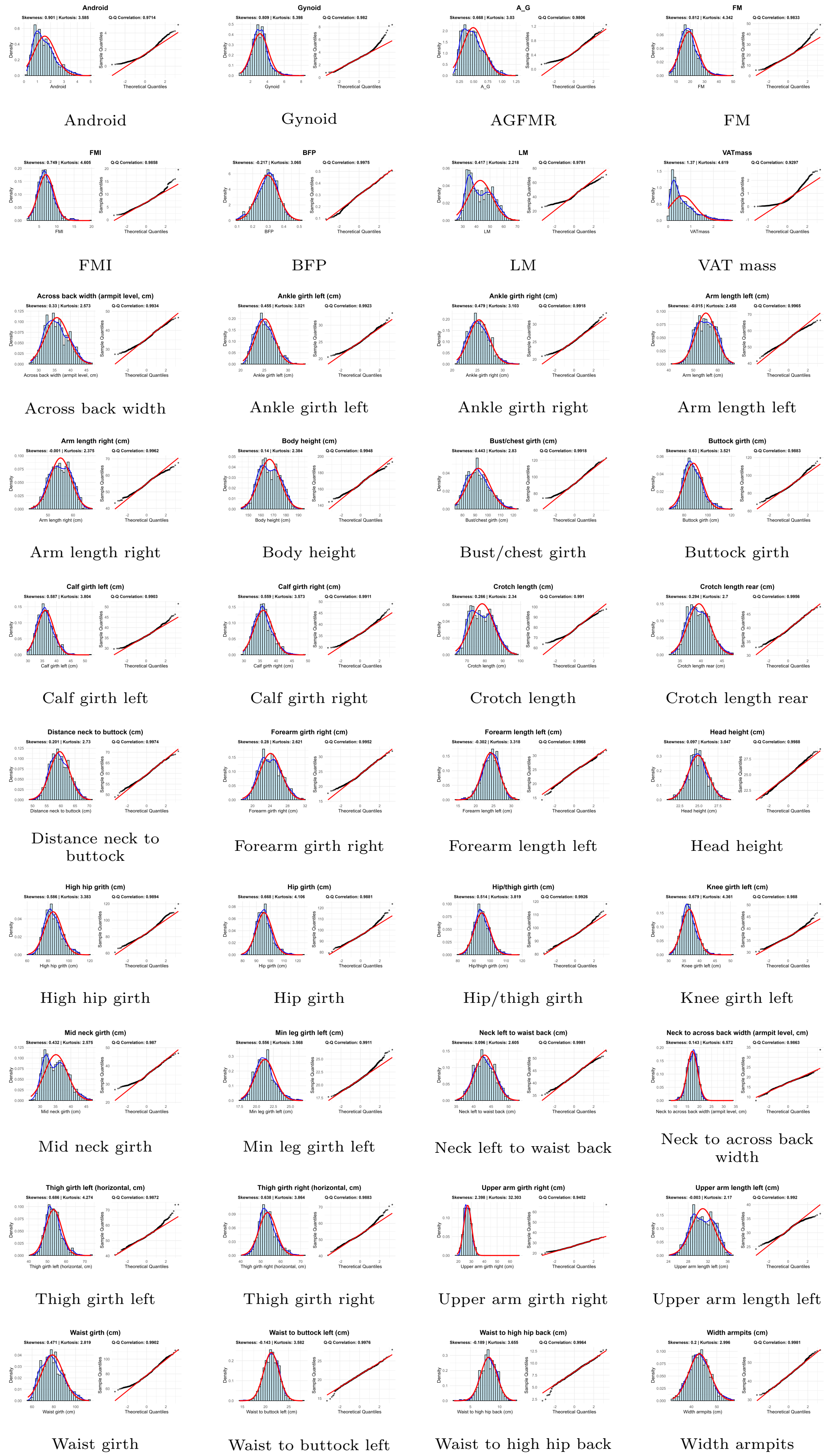

Fig. S 2: Quantile-Quantile (Q-Q) Plots and Histograms for main dependent and key independent variables.

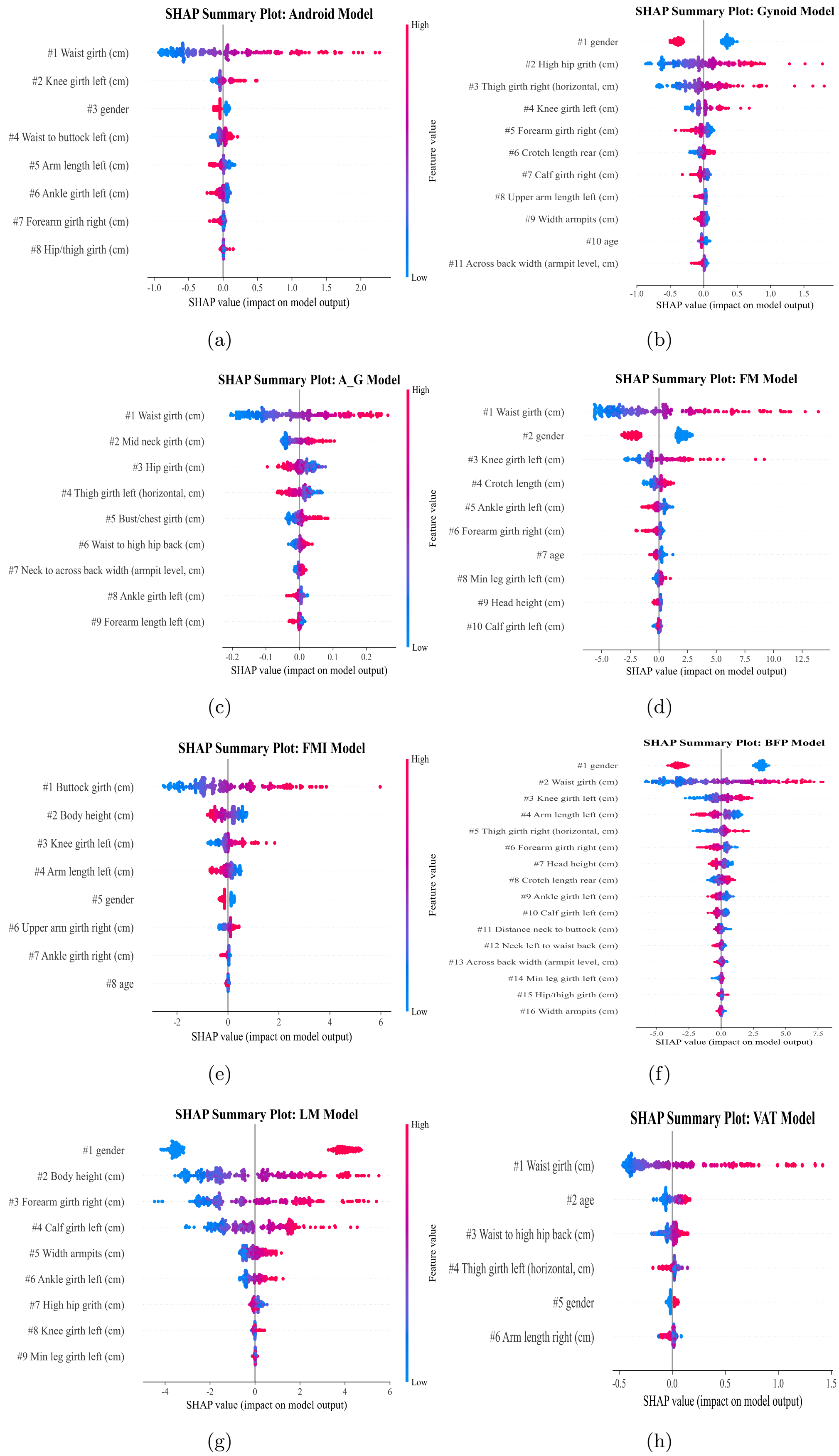

Fig. S 3: SHAP summary plot of the XGBoost model for predicting eight adiposity-related indices. The numerical prefix (#) indicates the overall feature rank.

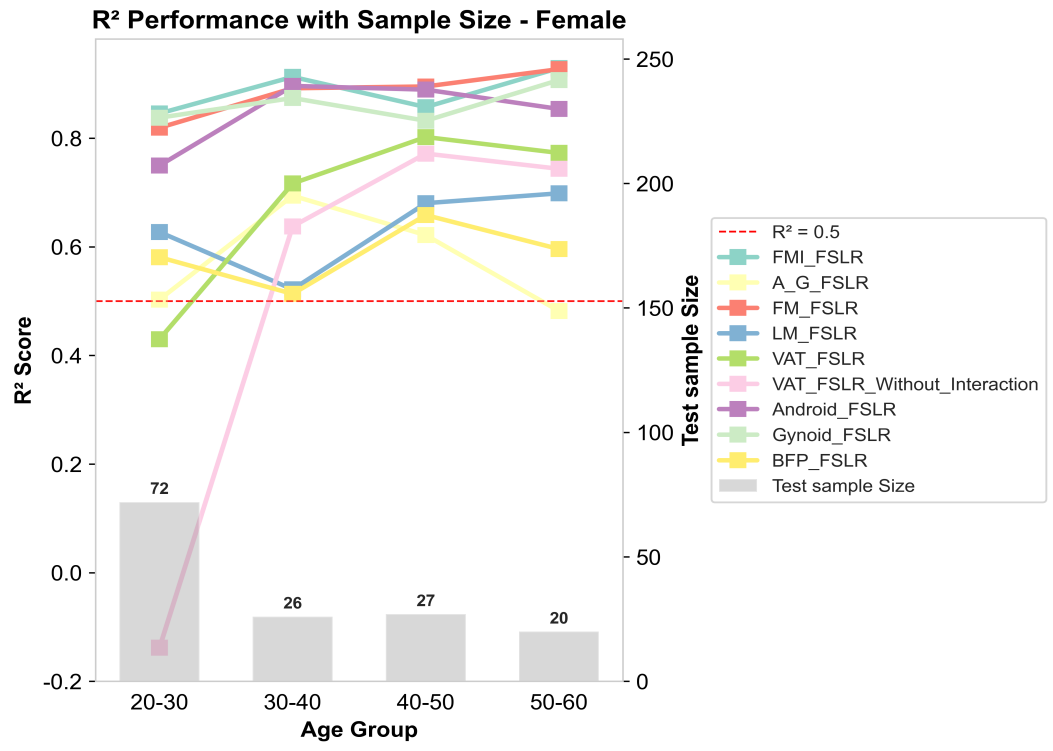

(a)

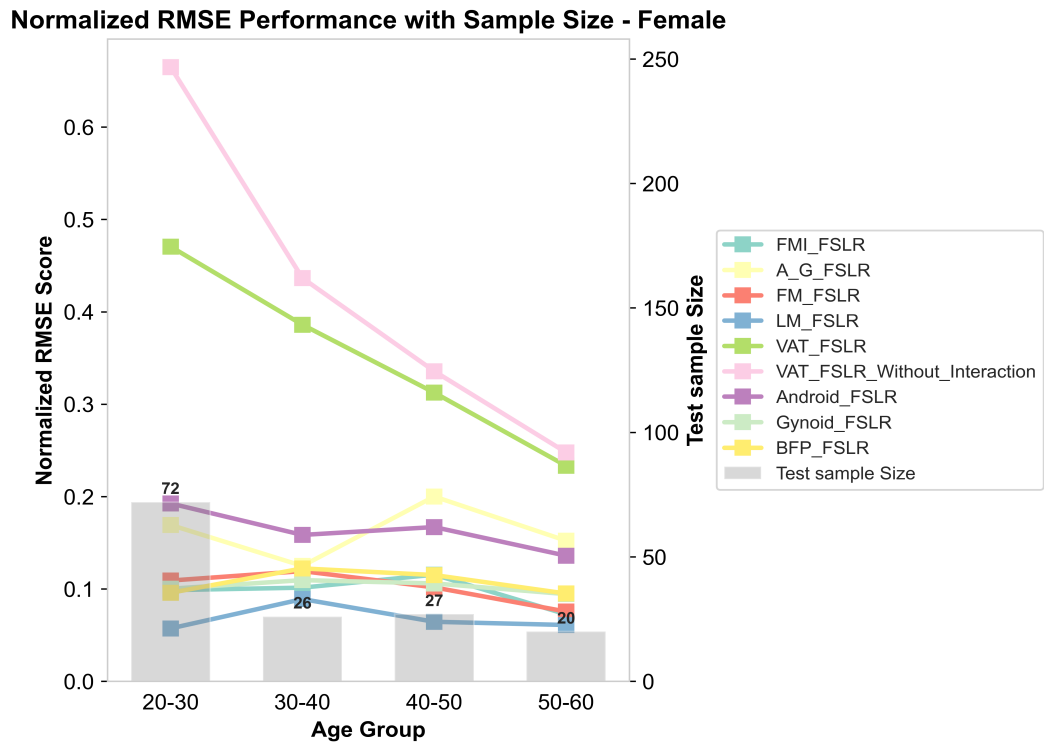

(b)

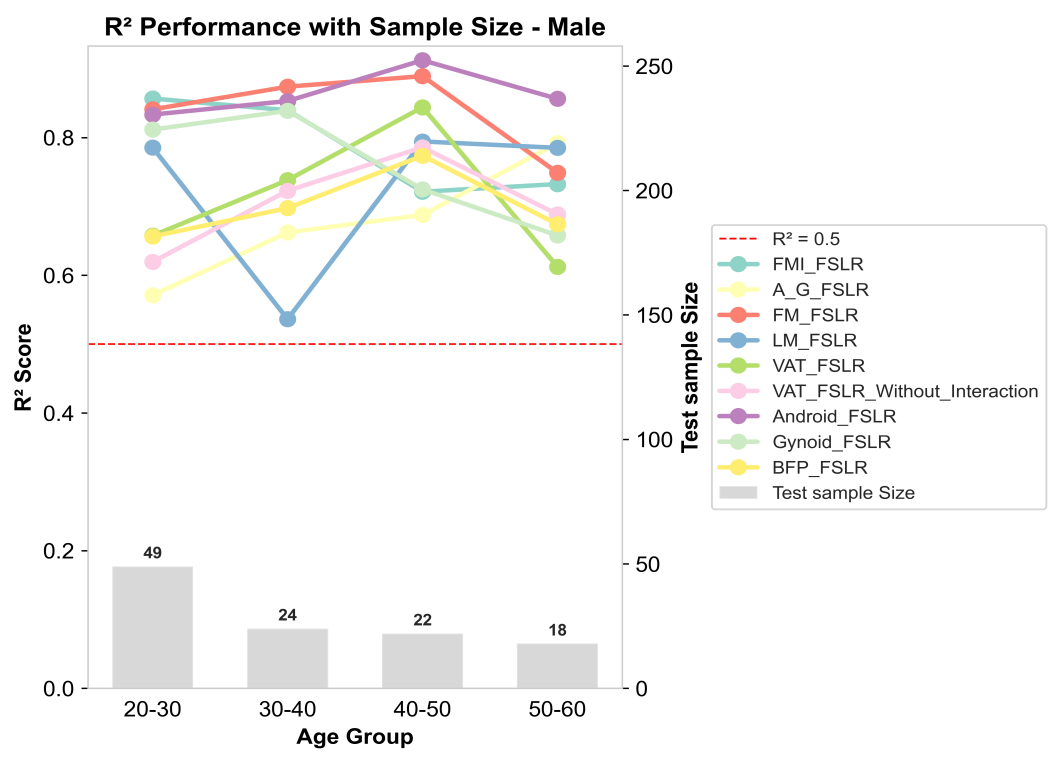

(c)

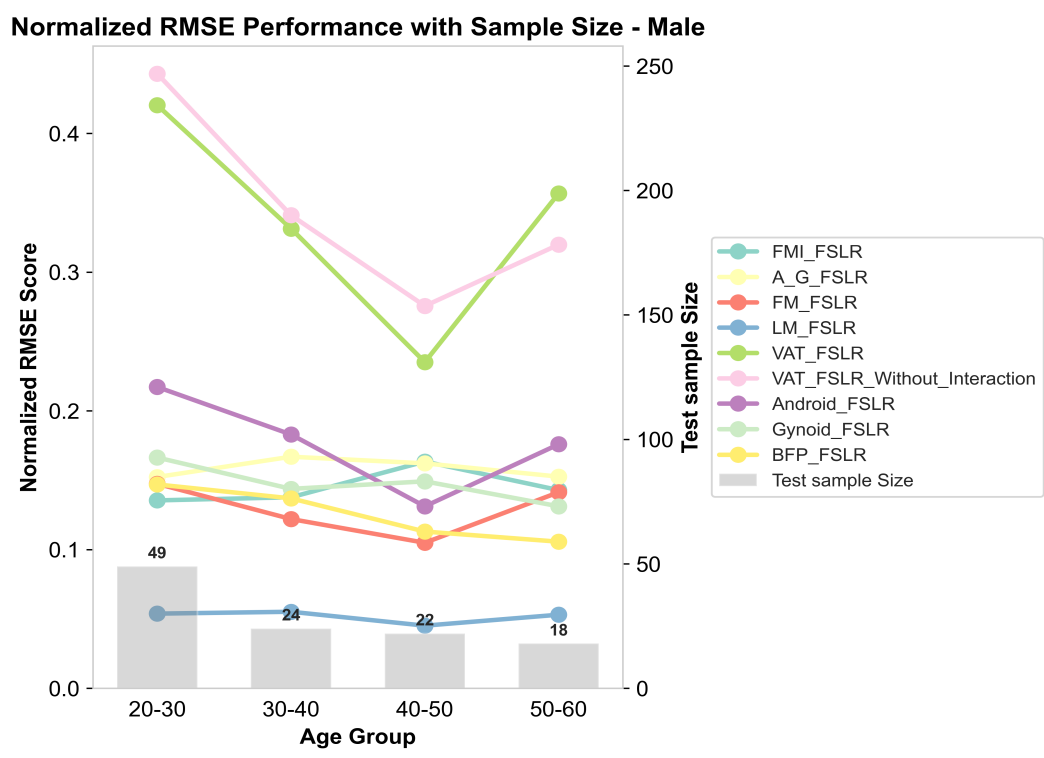

(d)

Fig. S 4: Model performance (R^2 and normalized RMSE) for FSLR models across age and sex subgroups.
